# High Insertion Torque—Clinical Implications and Drawbacks: A Scoping Review

**DOI:** 10.3390/medicina61071187

**Published:** 2025-06-30

**Authors:** Mattia Manfredini, Martina Ghizzoni, Beatrice Cusaro, Mario Beretta, Carlo Maiorana, Francisley Ávila Souza, Pier Paolo Poli

**Affiliations:** 1 Maxillofacial Surgery and Dental Unit, Fondazione IRCCS Cà Granda Policlinico, 20112 Milan, Italy; mattia.manfredini@unimi.it (M.M.); beatrice.cusaro@studenti.unimi.it (B.C.); carlo.maiorana@unimi.it (C.M.); pierpaolo.poli@unimi.it (P.P.P.); 2 Department of Biomedical, Surgical and Dental Sciences, University of Milan, Via della Commenda 10, 20122 Milan, Italy; 3 Department of Diagnosis and Surgery, School of Dentistry, São Paulo State University (UNESP), Araçatuba 16015-050, SP, Brazil; francisley.avila@unesp.br

**Keywords:** dental implant, high torque, implant failure, insertion torque, marginal bone loss, periimplantitis, primary stability

## Abstract

Implant primary stability is a prerequisite for obtaining osseointegration and clinical success. Insertion torque (IT) is measured during implant placement and is expressed in Ncm. It represents the quantification of the frictional force experienced by the implant as it progresses apically through a rotational motion along its axis. Usually, to achieve osseointegration, a value within the range of 20–40 Ncm is desirable. Below a threshold of 20 Ncm, implants have a decrease in survival rate, while implant stability is guaranteed above 20 Ncm. The main goal of this study was to evaluate whether high values of IT affect osseointegration, implant health, and healing, by highlighting the positive and negative effects of IT > 50 Ncm on peri-implant bone, soft tissues, and long-term stability. This scoping review considered randomized clinical trials, observational studies, and cohort studies. Studies failing to meet the predefined inclusion criteria were excluded from the analysis. The review process adhered to the Preferred Reporting Items for Scoping Reviews (PRISMA-ScR) guidelines. Ultimately, a total of 11 studies were included in the final synthesis. Based on the studies included, the literature suggests that high values of IT guarantee adequate primary stability and better osseointegration. However, high IT is significantly associated with greater marginal bone loss, depending on bone density. Accordingly, IT values > 50 Ncm may provoke greater compressive forces with a negative impact on the jawbone. An elevated strain on the bone can induce necrosis and ischemia, due to an alteration of circulation, which in turn is responsible for marginal bone loss and reduced osseointegration. Lack of osseointegration ultimately leads to an early implant failure. As concerns soft tissue recession, a higher decrease is measured in implants placed with high-insertion torque. Nonetheless, additional clinical trials are warranted to assess long-term outcomes, quantify the incidence of these complications, and explore the impact of emerging clinical variables.

## 1. Introduction

The introduction of dental implants gained momentum based on Brånemark’s concept of osseointegration, which he defined as “a direct structural and functional connection between the living bone and the surface of the load-carrying endosseous implant” [1]. The success of osseointegration is modulated by multiple factors, including minimally invasive surgical techniques, the application of surgical motors with torque and speed control, adequate cooling through sterile saline irrigation, and the use of biocompatible implant materials [1,2,3]. To ensure long-term success, two essential factors for osseointegration are primary stability and the absence of micromovements [4]. Primary stability (PS) of an implant is described as the level of stability observed immediately after its placement. Since it is closely related to the micro-movements of the implant, PS has traditionally been regarded as a key prognostic indicator for successful osseointegration and the overall success of the implant [5,6]. Primary stability (PS) can be assessed using various methods, including resonance frequency analysis (RFA) and insertion torque (IT) [2]. The present scoping review specifically focuses on the latter. IT is measured during the insertion of the implant and expressed in Ncm. IT represents the quantification of the frictional force experienced by the implant as it progresses apically through a rotational motion along its axis, and this is defined as the resistance to rotation at the moment of implant insertion [7,8]. For several years, elevated levels of IT were viewed as a critical factor for the successful osseointegration of implants, given its direct correlation with PS. Nevertheless, recent evidence demonstrated that reduced IT values can diminish bone tissue trauma, promoting or aiding the bone healing process without compromising PS [9,10,11]. Several factors influence insertion torque (IT), including bone density, the surgical technique used for implant site preparation, as well as the geometry and surface morphology of the implant [12,13,14]. High IT values were frequently found in dense bone (D1 type), whereas low values were detected in soft bone (D4) [15]. Another variable correlated to high IT is the under-preparation of the implant site [16,17,18,19,20]. There seems to be no unanimous consensus in the literature concerning the ideal IT values. Generally, to achieve osseointegration, a value within the range of 20–40 Ncm is desirable [6,12,21]. Below a threshold of 20 Ncm, implants have a decrease in survival rate [22,23]. Above 20 Ncm, implant stability is guaranteed [4]. In this respect, different data have been found in the literature. Clinical investigations agreed that torque values ranging from 20 to 30 Ncm suffice for dental implant osseointegration [4,24,25,26,27]. Several authors [13,23,28] hypothesized a positive correlation between IT and the consequent increase in PS. Although an IT > 50 Ncm could improve PS, it could also generate compression on peri-implant tissues [12,29]. Many clinical studies found the effect of high IT on marginal bone level, buccal soft tissue level, and implant failure. The main goal of this study was to evaluate if high values of IT could affect osseointegration, implant health, and healing by highlighting the positive and negative effects of IT > 50 Ncm on peri-implant bone, soft tissues, and long-term stability [12,13,15,29,30,31].

This review aims to analyze the clinical implications of high insertion torque on peri-implant soft and hard tissues.

### Focused Questions

May high insertion torque cause early marginal bone loss, soft tissue damage, and implant failure?

## 2. Materials and Methods

### 2.1. Eligibility Criteria

The inclusion criteria established for this review encompassed the following: (I) study design—interventional studies and observational studies; (II) patients undergoing implant placement; (III) interventions—implant placement with high values of insertion torque; and (IV) outcome—clinical results of implant placed with high torque. The analysis was restricted to studies that fully met the predefined inclusion criteria. Exclusion criteria encompassed the following: (I) abstracts or articles published in languages other than English; (II) duplicate publications; (III) studies considered irrelevant to the research objectives, including those investigating unrelated adjunctive therapies or inconsistent with the abstract’s content; (IV) ex vivo or animal experimental studies; (V) studies lacking ethics committee approval; and (VI) narrative reviews, systematic reviews, or meta-analyses.

### 2.2. Search Strategy

A three-stage search strategy was employed in accordance with the Joanna Briggs Institute (JBI) guidelines for scoping reviews. The initial phase involved a preliminary search conducted in PubMed (MEDLINE) and Scopus using defined parameters. In the second phase, key terms and concepts were extracted from the literature identified during the exploratory search to develop a refined and comprehensive search strategy. In the final phase, the reference lists of all included studies were reviewed to identify any additional relevant publications [32].

Additionally, the Population–Concept–Context (PCC) framework was utilized to guide the review. This framework comprises three core elements: the population (patients receiving dental implants), the concept (application of high insertion torque during implant placement), and the context (regardless of cultural or environmental setting). Examination of abstracts from studies investigating various suture materials used in third molar surgeries was conducted. Throughout this extensive literature review, adherence to the preferred reporting items for scoping reviews (PRISMA-ScR) consensus was upheld, as outlined in Table S1 [33].

### 2.3. Research

Medical Subject Headings (MeSH) employed in the search strategy included terms such as “torque”, “dental implants”, “bone resorption”, “peri-implantitis”, and “complications”. A comprehensive electronic search was conducted across PubMed (MEDLINE) and Scopus databases. Studies published between 2011 and 2024 were considered eligible, with data extraction performed from February to May 2024. The final search was completed on 16 September 2023.

Two independent reviewers (M.G. and B.C.) conducted the screening and selection process. Discrepancies between reviewers were resolved through discussion and consensus. In cases of uncertainty or complexity, additional input was sought from three other reviewers (P.P.P., C.M., M.M., M.B., and F.A.S.). The screening process began with a title and abstract evaluation to exclude non-relevant studies. Subsequently, full-text assessments were conducted to determine eligibility based on the established inclusion criteria. Outcomes of interest were carefully recorded, and only studies that met all inclusion criteria were retained for the final synthesis.

This review protocol was prospectively registered on the Open Science Framework (OSF) platform (Registration, accessed on 30 April 2025, https://osf.io/ek2tz). The complete search strategies applied for each database are detailed in Table S2.

### 2.4. Quality Assessment of Included Studies

In the present investigation, the risk of bias in clinical studies was evaluated through a qualitative approach based on the National Heart, Lung, and Blood Institute (NHLBI) Quality Assessment Tools for Controlled Intervention Studies and for Observational Cohort and Cross-Sectional Studies. This methodological framework enabled a comprehensive and systematic appraisal of the methodological quality and potential biases within the included studies, thereby supporting the assessment of the credibility and validity of the synthesized findings [34].

## 3. Results

**Figure 1:**
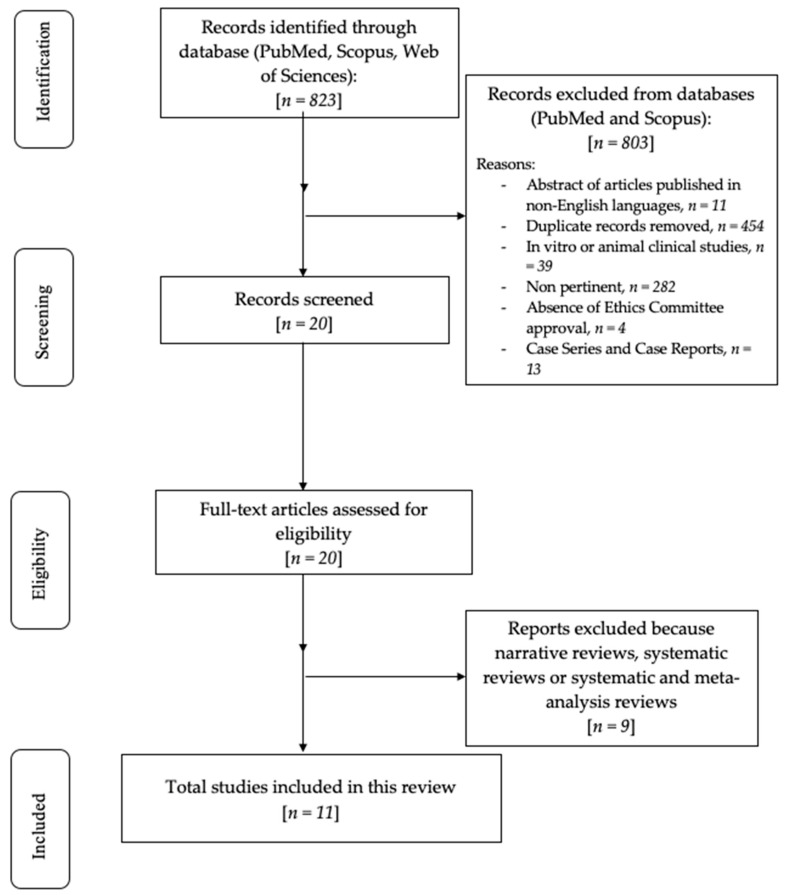
Flowchart of the review process.

The initial database search, based on MeSH terms, yielded a total of 823 articles. Following the application of exclusion criteria, 803 studies were removed: 11 due to publication in languages other than English, 454 as duplicates, 39 for being in vitro or animal studies, 282 for lacking relevance to the research objectives, 4 due to the absence of Ethics Committee approval, and 13 for being case reports or case series. As a result, 20 articles were retained for further evaluation based on their titles and abstracts. Moreover, 9 articles were excluded since irrelevant. The remaining 11 articles were assessed for eligibility, included, and scrutinized for this review. The flowchart of the review process is described in Figure 1.

Table S3 (Supplementary Materials) shows the studies excluded from this review and the reasons for exclusion. The studies belonged to two categories: controlled intervention studies [29,30,31,35] and observational cohort studies [12,15,27,36,37,38,39].

### Risk of Bias

The Cochrane Collaboration tool was utilized to assess the risk of the randomized clinical trials selected for this review (Table 1). The risk of bias for non-randomized clinical studies included was assessed through the ROBINS-I tool (Table S4). Table S5 shows the risk of bias of the studies included in this review through the ROBINS-I assessment tool.

A low risk of bias was observed in this review.

Table 2 shows the baseline characteristics of patients included in the selected studies. Evidence of studies included in this review (study design and aim, methods, results, and conclusions) is shown in Table S6 (Supplementary Materials).

The quality assessment of controlled intervention studies was conducted using the NHLBI Quality Assessment Tool; the results of which are presented in Table S7 (Supplementary Materials). Similarly, the evaluation of observational cohort and cross-sectional studies was performed using the corresponding NHLBI tool, with outcomes detailed in Table S8 (Supplementary Materials).

Table 3 summarizes the data extracted from the 11 articles included in the review, focusing on marginal bone loss (MBL), facial soft tissue level (FSTL), and implant failure. The table provides a concise overview of the main findings, facilitating comparison across studies and highlighting relevant trends and outcomes.

## 4. Discussion

Eleven studies were selected for this scoping review and categorized into two groups: controlled intervention studies and observational/cohort studies. These studies explored various complications associated with elevated insertion torque (IT) values, including marginal bone loss (MBL), facial soft tissue level (FSTL) recession, and implant failure [29,33].

### 4.1. Marginal Bone Loss and Implant Failure

Marginal bone loss is one of the primary complications following implant placement. Changes in peri-implant bone levels are commonly linked to bacterial infection, surgical trauma, occlusal overload, patient-related variables, and implant design factors such as the collar or neck configuration [31,34,38,40]. Implant failure is defined by implant mobility and non-physiological peri-implant bone loss and may present with pain, discomfort, altered sensations, and infection [29,35].

### 4.2. Insertion Torque and Primary Stability

Insertion torque (IT), measured in Ncm, is widely used as a proxy for primary stability (PS) at the time of implant placement [2,12,35]. While consensus exists on the importance of achieving adequate PS, the optimal IT range for successful osseointegration remains debated [12]. Several studies recommend an IT range between 20 and 40 Ncm to prevent micromovements and ensure stability [12]. Excessive micromovements (beyond 150 µm) are frequently associated with implant failure, as they can interfere with early healing and osseointegration [34,41]. High IT values are thought to enhance primary stability and improve clinical outcomes [13,35]. However, values exceeding the jawbone’s elastic limit may induce micro-cracks and increase the risk of crestal bone loss [10]. Bone can tolerate strain within its elastic threshold due to its viscoelastic properties, but exceeding this threshold may result in microfractures, ischemic necrosis, and bone resorption [30].

### 4.3. Impact of IT on Bone Compression and Necrosis

Elevated IT values can lead to excessive bone compression and localized stress, potentially delaying healing and causing ischemia [16,18,35,42,43]. A direct correlation exists between increasing IT and the likelihood of compression necrosis. Conversely, lower IT values reduce stress on the peri-implant bone [41]. Most included studies compared implants placed with regular IT (<50 Ncm) and high IT (>50 Ncm). The appropriate torque level is often influenced by bone density: D1 (dense bone) typically requires higher IT, whereas D4 (soft bone) corresponds with lower IT values. Intermediate IT values are observed in D2–D3 bone types. Statistically significant correlations have been observed between bone volume and insertion torque [12,14,15].

### 4.4. Marginal Bone Loss: Comparative Findings

MBL remains one of the most critical complications in implantology [44,45]. It is commonly assessed via intraoral radiographs by measuring the vertical distance from the implant apex to a reference point on adjacent bone. In the mandible, implants placed with regular IT (<50 Ncm) showed a mean MBL of 1.03 ± 0.12 mm, compared to 1.53 ± 0.29 mm for those with high IT (>50 Ncm), a highly significant difference (*p* < 0.001) over a 3-year follow-up [41]. Similarly, in the maxilla, MBL was 0.96 ± 0.46 mm for the regular IT group and 1.16 ± 0.61 mm for the high IT group (*p* < 0.0001) [35]. These results indicate that higher IT values are consistently associated with increased marginal bone loss, regardless of the jaw region. Furthermore, one of the studies highlights a significant distinction between thin and thick gingival biotypes in relation to marginal bone loss. Specifically, in the study there were 31 patients (83.8%) exhibited a thick biotype, while only 6 patients (16.2%) presented with a thin biotype. The mean marginal bone loss (MBL) recorded from implant placement to crown delivery was 0.9 mm (±0.18 mm; range: 0.6–1.3 mm), increasing to 1.21 mm (±0.19 mm; range: 0.8–1.5 mm) at the 1-year follow-up. Patients with a thin gingival biotype demonstrated significantly greater bone loss compared to those with a thick biotype at both time points (*p* < 0.05). These findings suggest that soft tissue thickness plays a critical role in the maintenance of peri-implant bone levels [38].

### 4.5. High Insertion Torque and Soft Tissue Level

Facial soft tissue level (FSTL) is a critical variable in assessing peri-implant health [46]. It is typically measured by evaluating the discrepancy between the facial gingival margin of the implant and the adjacent natural tooth [35]. Peri-implant mucosal recession has been observed in both low (<35 Ncm) and high insertion torque (IT) groups, though more pronounced in implants placed with higher IT. At 36 months post-implantation, FSTL measurements indicated a minimal change (0.03 mm) in the regular IT group in the maxilla, compared to a significant recession of −0.54 mm in the high-IT group (*p* < 0.001). Similarly, in the mandible, recession values were −0.26 mm for regular IT and −1.40 mm for high IT (*p* < 0.001) [35]. This soft tissue recession appears to be influenced by buccal bone thickness (BBT), especially after implant site preparation. Implants placed in sites with a BBT < 1 mm showed greater soft tissue loss. Conversely, sites with BBT > 1 mm demonstrated significantly less FSTL reduction [30].

### 4.6. High Insertion Torque and Implant Failure

High insertion torque has also been associated with a greater incidence of peri-implantitis, a leading cause of implant failure [30,47,48,49]. Implant failure is typically characterized by mobility, instability, progressive bone loss, or the necessity for implant removal due to infection [29,35]. There are two key metrics to evaluate implant outcomes: survival rate, referring to the physical presence of the implant in the oral cavity, and success rate, which takes into account clinical parameters. According to Buser’s criteria, success requires that marginal bone loss does not exceed 1.5 mm in the first year and remains under 0.2 mm annually thereafter [35,50]. Interestingly, implant failures in the reviewed literature were primarily restricted to dense (D1) and soft (D4) bone types, with medium-density bone (D2–D3) offering a more favorable prognosis and being considered a “safer zone” for implantation [15,51].

### 4.7. High Insertion Torque and Osseointegration

Clinically, implants placed with IT values above 50 Ncm have shown increased marginal bone loss and greater facial soft tissue recession. These outcomes are often attributed to excessive compressive forces exerted on the jawbone, which may exceed its physiological stress threshold, causing microcracks and compromising implant stability [52]. High IT can also delay healing, hinder osseointegration, and elevate the risk of bone resorption [30,35,53,54,55,56,57]. Excessive pressure impairs microcirculation and angiogenesis, leading to ischemia, reduced osteogenesis, and eventual necrosis of peri-implant bone [35,48]. An ideal osseointegration process involves minimal micromovements, low compressive stress, and limited bone remodeling [29]. While high IT is often correlated with improved primary stability (PS) [13,49,58], beyond a certain threshold, it may induce harmful biomechanical strain, disrupting circulation and contributing to bone necrosis and implant instability [49,59]. Additionally, differences in bone density between the maxilla and mandible may influence outcomes. D1-type bone, often found in the mandible, is more prone to crestal bone loss due to higher compressive forces [12,30]. Nevertheless, not all studies concur on the detrimental effects of high IT. Some findings suggest that while high IT enhances PS, it does not necessarily correlate with increased marginal bone loss or impaired osseointegration. These studies argue that elevated IT does not negatively impact implant survival or success rates [27,60,61,62].

In addition, histological evidence presented by Campos et al. indicates that elevated insertion torque during implant placement induces significant compression-related bone necrosis [63]. In contrast, when lower torque levels are applied, both necrotic changes and subsequent bone remodeling are considerably reduced. Furthermore, reduced mechanical stress appears to promote the development of healing chambers characterized by intramembranous-like bone, which may facilitate a more rapid progression toward secondary implant stability [64].

### 4.8. Limitations and Future Perspectives

This report acknowledges several limitations. The search strategy adopted may have been overly specific, given the broad nature of a scoping review, potentially limiting the inclusion of relevant studies. Furthermore, the comparability of outcomes across the included studies may be constrained by variability in study populations and methodological heterogeneity. Indeed, clinical variables may differ due to the sample size considered or the capabilities of clinicians involved. Comparable limitations pertain to surgical techniques and clinical management, both of which may differ depending on the clinician’s level of expertise and the patient’s adherence to postoperative care protocols. Furthermore, the diversity of implants on the market can impact clinical outcomes and, consequently, the results of clinical trials. Future investigations, particularly randomized clinical trials, are essential for a thorough examination of high insertion torque and its consequences on marginal bone loss, soft tissue levels, and implant failure. Moreover, research should explore longer follow-up periods and other variables related to high insertion torque [65,66]

## 5. Conclusions

The review highlights a correlation between an increased number of implants and a rising incidence of postoperative complications related to high values of insertion torque (IT). These complications include peri-implant marginal bone loss (MBL), recession of facial soft tissue level (FSTL), and implant failure. High-IT values (>50 Ncm) can negatively impact osseointegration, increase bone compression, lead to marginal bone loss, and contribute to implant failure [67,68]. Most of the selected articles showed a greater MBL, FST recession, implant failure, and diminished osseointegration in implants placed with a torque value > 50 Ncm.

## Figures and Tables

**Table 1:** Risk of bias of the randomized clinical studies included in this review: the green symbol represents a low risk of bias, while the yellow symbol represents a high risk of bias.

References (Authors, Year of Publication)	Random Sequence Generation	Allocation Concealment	Blinding	Incomplete Outcome Data	Selective Reporting
Barone et al., 2015 RCT [29]	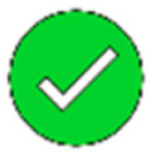	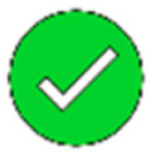	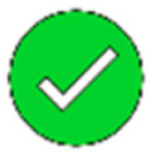	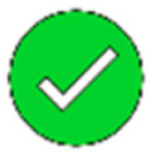	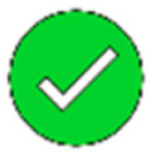
Alfonsi et al., 2016 RCT [30]	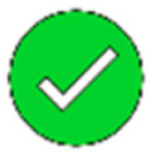	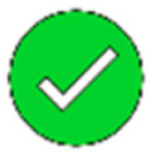	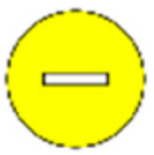	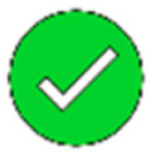	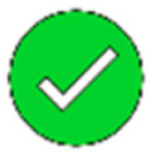
Hof et al., 2014 RCT [31]	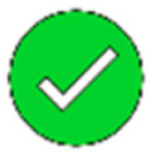	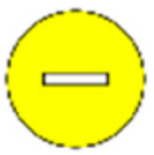	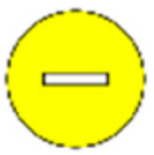	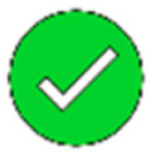	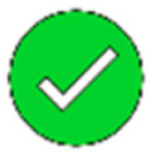
Marconcini et al., 2018 RCT [35]	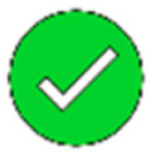	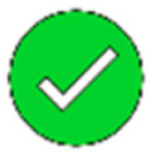	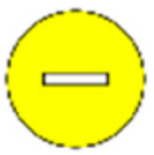	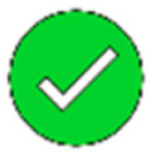	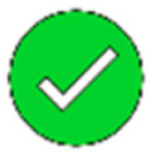

**Table 2:** Baseline characteristics of patients selected in the included studies.

References (Authors, Year of Publication, and Study Design)	N° of Patients and % Women	Mean Age (Years), Mean (SD or Range)	Inclusion and Exclusion Criteria	Torque Value
Aldahlawi et al., 2018 Retrospective study [12]	66 46.9% Fe	56.31 ±16.20 (range 28–84)	Inclusion criteria: patients who received at least one implant that had been in function for an average post-loading period of 12 months and availability of post-loading radiographic and clinical follow-up, partially edentulous.	Regular-IT group: 37.9 ± 12.62 Ncm High-IT group: 67.35 ± 4.0 Ncm
Makary et al., 2011 XX [15]	18 61.1% Fe	48.8 ± 13.8 (range 30–74)	Inclusion criteria: good health, with no systemic disorders, and able to sign an informed consent form.	IT within the range: 15–150 Ncm (mean value 78.30 Ncm)
Rizkallah et al., 2013 Retrospective study [27]	145 57.2% Fe	57.5	Inclusion criteria: N.R. Exclusion criteria: N.R.	Mean IT 72.0 Ncm (range 23.8–178 Ncm)
Barone et al., 2015 RCT [29]	116 66.4% Fe	51.4 ± 8.1	Inclusion criteria: able to sign an informed consent form, partially edentulous, required at least one single implant, older than 18 years Exclusion criteria: history of systemic diseases that would contraindicate oral surgical treatment, long-term non-steroidal anti-inflammatory drug therapy, lack of opposite occluding dentition in the area intended for implant supported restoration, extraction sites with less than 3 months of healing, presence of severe untreated periodontal disease, bone augmentation required at the time of implant placement, poor oral hygiene and compliance (presence of stain, calculus and plaque before dental implant surgery), pregnancy or nursing, unwillingness to return for the follow-up examination, use of more than 10 cigarettes per day (subject smoking <10 cigarettes per day were required to stop smoking before and after surgery).	Regular-IT group: 30.3 ± 7.5 Ncm High-IT group: 68.8 ± 9.0 Ncm
Alfonsi et al., 2016 RCT [30]	116 66.4% Fe	51.4 ± 8.1	Including criteria: able to sign an informed consent form, partially edentulous, required to have at least one single implant, and older than 18 years. Exclusion criteria: history of systemic diseases that would contraindicate oral surgical treatment, long-term non-steroidal anti-inflammatory drug therapy, lack of opposite occluding dentition in the area intended for implant supported restoration, extraction sites with less than 3 months of healing, presence of severe untreated periodontal disease, poor oral hygiene and compliance (presence of stain, calculus and plaque before dental implant surgery), pregnancy or nursing, unwillingness to return for the follow-up examination, use of more than 10 cigarettes per day (subject smoking <10 cigarettes per day were required to stop smoking before and after surgery).	Regular-IT group: 30.3 ± 7.5 Ncm High-IT group: 68.8 ± 9.0 Ncm
Hof et al., 2014 RCT [31]	21 61.9% Fe	67.4 (range 45–86)	Inclusion criteria: edentulous mandibles, teeth extracted for at least 6 months, sufficient bone volume in height and width to allow implant placement, without any augmentation procedure. Exclusion criteria: any medical or psychiatric contraindication to implant surgery.	Low-IT group: ≤20 Ncm High-IT group: >50 Ncm
Marconcini et al., 2018 RCT [35]	116 66.4% Fe	51.4 ± 8.1	Inclusion criteria: able to sign an informed consent form, partially edentulous, required at least one single implant, older than 18 years. Exclusion criteria: history of systemic diseases that would contraindicate oral surgical treatment, long-term non-steroidal anti-inflammatory drug therapy, lack of opposite occluding dentition in the area intended for implant-supported restoration, recent (<10 years) or ongoing intravenous/oral bisphosphonate therapy, extraction sites with less than 3 months of healing, presence of severe untreated periodontal disease, bone augmentation required at the time of implant placement, poor oral hygiene and motivation, pregnancy or nursing, unwillingness to return for the follow-up examination, use of more than 10 cigarettes per day (subject smoking <10 cigarettes per day were required to stop smoking before and after surgery).	Regular-IT group: 20–50 Ncm High-IT group: 50–100 Ncm
Bidgoli et al., 2015 Retrospective cohort study [36]	136 57.4% Fe	Age within the range 21–69	Exclusion criteria: systemic disease or conditions such as controlled diabetes, osteoporosis and history of radiotherapy or chemotherapy, pregnancy or nursing, consuming corticosteroids 4 months before surgery, smoking, substance abuse, consumption of alcoholic beverages, performing bone augmentation or sinus elevation protocol during implant insertion, periodontal disease, complication in healing after implant surgery.	Low-IT group: 20–30 Ncm High-IT group: 45–70 Ncm
Khayat et al., 2011 Prospective study [37]	38 60.5% Fe	Regular-IT group: 63 (range 34–75) High-IT group: 64 (range 32–84)	Inclusion criteria: exhibited adequate oral hygiene and expressed a firm commitment to follow-up visits, >18 years, 1 or more missing teeth in either jaw, ability and willingness to comply with all study requirements, available for clinical follow-up, sufficient bone volume with or without localized bone grafting to accommodate implants at least 10 mm in length, absence of clinical or systemic conditions that would contraindicate surgery, implant placement, and/or implant survival. Accessibility for insertion torque measurement device. Exclusion criteria: heavy smoking (>20 cigarettes daily), alcohol or drug abuse, infection, endodontic or periodontal problems in teeth adjacent to the implant site, extraction sites with less than 6 months of healing, general pathologies or contraindications for implant treatment or surgery.	Regular-IT group: 30–50 Ncm High-IT group: 50–70 Ncm
Oskouei et al., 2023 Prospective cohort study [38]	37 45.9%	49.16 ± 9.42 (range 35–70)	Inclusion criteria: did not have a specific systemic problem interfering with the surgical process and osseointegration, did not have a history of taking bisphosphonates, volume and quality of the bone sufficient for placing the implant, no need for bone grafting at the implant placement site, had a missing tooth area in the posterior region of the mandible that had been extracted for at least 6 months, had the ability and willingness to participate in the 1-year follow-up. Exclusion criteria: had a systemic problem interfering with the surgical process and osseointegration, had a history of taking bisphosphonates or was currently taking it, had a history of head and neck radiotherapy, volume and quality of the bone were not enough to place the implant, bone augmentation was required at the time of implant placement, had parafunctional habits such as bruxism and clenching, there were infection and endodontal and periodontal problems around the implant placement, there was no natural dentition in the opposite jaw.	20–40 Ncm (mean value 28.32 Ncm)
Grandi et al., 2012 Cohort study [39]	102 62.7% Fe	Regular-IT group: 55.3 (range 43–67) High-IT group: 51.8 (range 39–65)	Inclusion criteria: at least 18 years, sufficient bone volume for placement of implants at least 8 mm in length and 3.7 mm in diameter, healed bone sites (at least 4 months post-extraction), adequate oral hygiene, plaque index ≤2. Exclusion criteria: systemic disease that could compromise osseointegration, previous irradiation in the head and neck area, treatment or under treatment with intravenous amino-bisphosphonates, uncontrolled diabetes, substance abuse, heavy smoking (>20 cigarettes daily), acute or chronic infection/inflammation in the area intended for implant placement.	Regular-IT group: 30–45 Ncm High-IT group: 50–80 Ncm

**Table 3:** MNL, FSTL, and implant failure values found in the selected articles.

Author	MBL	FSTL	Implant Failure
Aldahlawi et al., 2018 [12]	Regular-IT group: 0.18 ± 0.68 High-IT group: 0.95 ± 1.60		Survival rate: 94.6%
Makary et al., 2011 [15]			
Rizkallah et al., 2013 [27]			
Barone et al., 2016 [29]	Regular-IT group: Maxilla: −0.55 ± 0.37 mm Mandible: −0.36 ± 0.31 mm High-IT group: Maxilla: −0.88 ± 0.43 mm Mandible: −1.23 ± 0.36 mm	Regular-IT group: Maxilla: −0.07 ± 0.38 mm Mandible: −0.57 ± 0.50 mm High-IT group: Maxilla: −0.13 ± 0.34 mm Mandible: −0.90 ± 0.48 mm	Survival rate: 97.4%
Alfonsi et al., 2016 [30]	Regular-IT group: Maxilla: −0.67 ± 0.43 mm Mandible: −0.75 ± 0.28 mm High-IT group: Maxilla: −0.93 ± 0.57 mm Mandible: −1.31± 0.33 mm	Regular-IT group: Maxilla: −0.10 ± 0.49 mm Mandible: −0.13 ± 0.34 mm High-IT group: Maxilla: −0.62 ± 0.57 mm Mandible: −1.13 ± 0.50 mm	Regular-IT group survival rate: 98.2% High-IT group survival rate: 94.8%
Hof et al., 2014 [31]	Low-IT group: 0.69 mm High-IT group: 0.68 mm		
Marconcini et al., 2018 [35]	Regular-IT group: Maxilla: 0.96 ± 0.46 mm Mandible: 1.03± 0.12 mm High-IT group: Maxilla: 1.16 ± 0.61 mm Mandible: 1.53 ± 0.29 mm	Regular-IT group: Maxilla: 0.03 mm Mandible: −0.26 mm High-IT group: Maxilla: −0.54 mm Mandible: −1.40 mm	Survival rate: 96.5%
Bidgoli et al., 2015 [36]			
Khayat et al., 2011 [37]			
Oskouei et al., 2023 [38]	1.01 ± 0.15 mm		
Grandi et al., 2012 [39]	Regular-IT group: 0.45± 0.25 High-IT group: 0.41 ± 0.18		

## Data Availability

Upon request to the corresponding author, the data are available for use. The protocol of the review was registered with the Open Science Framework (OSF) at https://osf.io/ek2tz (accessed on 30 April 2025).

## Supplementary Materials

The following supporting information can be downloaded at https://www.mdpi.com/article/10.3390/medicina61071187/s1, Table S1: PRISMA-ScR checklist; Table S2: Search strategies for electronic databases; Table S3: Summary table of studies excluded in this scoping review; Table S4: Criteria for judging risk of bias in ROBINS-I assessment tool; Table S5: Risk of bias of the studies included in this review through ROBINS-I assessment tool; Table S6: Evidence of studies included in this scoping review; Table S7: NHLBI quality assessment tool for controlled intervention studies; Table S8: NHLBI quality assessment tool for observational cohort and cross-sectional studies.
