# Supplementary material for: High Insertion Torque—Clinical Implications and Drawbacks: A Scoping Review
Source: Medicina (Kaunas). 2025 Jun 30;61(7):1187. doi: 10.3390/medicina61071187 (PMC12297995; doi:10.3390/medicina61071187)
Supplement: Supplementary file 1 [file medicina-61-01187-s001.zip › medicina-3700628-supplementary.pdf]

Table S1. PRISMA-ScR Checklist.

SECTION	ITEM	PRISMA-ScR CHECKLIST ITEM	REPORTED ON PAGE #
**TITLE**			
Title	1	Identify the report as a scoping review.	Title
**ABSTRACT**			
Structured summary	2	Provide a structured summary that includes (as applicable): background, objectives, eligibility criteria, sources of evidence, charting methods, results, and conclusions that relate to the review questions and objectives.	Abstract
**INTRODUCTION**			
Rationale	3	Describe the rationale for the review in the context of what is already known. Explain why the review questions/objectives lend themselves to a scoping review approach.	Initial introduction
Objectives	4	Provide an explicit statement of the questions and objectives being addressed with reference to their key elements (e.g., population or participants, concepts, and context) or other relevant key elements used to conceptualize the review questions and/or objectives.	End of introduction
**METHODS**			
Protocol and registration	5	Indicate whether a review protocol exists; state if and where it can be accessed (e.g., a Web address); and if available, provide registration information, including the registration number.	Dedicated section in M&M
Eligibility criteria	6	Specify characteristics of the sources of evidence used as eligibility criteria (e.g., years considered, language, and publication status), and provide a rationale.	Dedicated section in M&M
Information sources*	7	Describe all information sources in the search (e.g., databases with dates of coverage and contact with authors to identify additional sources), as well as the date the most recent search was executed.	Dedicated section in M&M
Search	8	Present the full electronic search strategy for at least 1 database, including any limits used, such that it could be repeated.	Dedicated section in M&M
Selection of sources of evidence†	9	State the process for selecting sources of evidence (i.e., screening and eligibility) included in the scoping review.	Dedicated section in M&M
Data charting process‡	10	Describe the methods of charting data from the included sources of evidence (e.g., calibrated forms or forms that have been tested by the team before their use, and whether data charting was done independently or in duplicate) and any processes for obtaining and confirming data from investigators.	Dedicated section in M&M
Data items	11	List and define all variables for which data were sought and any assumptions and simplifications made.	Dedicated

SECTION	ITEM	PRISMA-ScR CHECKLIST ITEM	REPORTED ON PAGE #
			section in M&M
Critical appraisal of individual sources of evidence§	12	If done, provide a rationale for conducting a critical appraisal of included sources of evidence; describe the methods used and how this information was used in any data synthesis (if appropriate).	Dedicated section in M&M
Synthesis of results	13	Describe the methods of handling and summarizing the data that were charted.	Dedicated section in M&M
**RESULTS**			
Selection of sources of evidence	14	Give numbers of sources of evidence screened, assessed for eligibility, and included in the review, with reasons for exclusions at each stage, ideally using a flow diagram.	Dedicated table
Characteristics of sources of evidence	15	For each source of evidence, present characteristics for which data were charted and provide the citations.	Dedicated table
Critical appraisal within sources of evidence	16	If done, present data on critical appraisal of included sources of evidence (see item 12).	Dedicated table
Results of individual sources of evidence	17	For each included source of evidence, present the relevant data that were charted that relate to the review questions and objectives.	Dedicated table
Synthesis of results	18	Summarize and/or present the charting results as they relate to the review questions and objectives.	Dedicated table
**DISCUSSION**			
Summary of evidence	19	Summarize the main results (including an overview of concepts, themes, and types of evidence available), link to the review questions and objectives, and consider the relevance to key groups.	Followed
Limitations	20	Discuss the limitations of the scoping review process.	Followed
Conclusions	21	Provide a general interpretation of the results with respect to the review questions and objectives, as well as potential implications and/or next steps.	Followed
**FUNDING**			
Funding	22	Describe sources of funding for the included sources of evidence, as well as sources of funding for the scoping review. Describe the role of the funders of the scoping review.	None

Table S2: Search strategies for electronic databases.

Database	Search strategy
PubMed (MEDLINE)	#1 “Torque” [MESH] OR (Torques)<br><br>#2 “Dental Implants ” [MESH] OR (Implant, Dental) OR (Implants, Dental) OR (Dental Implants, Mini) OR (Dental Implant, Mini) OR (Mini Dental Implant) OR (Mini Dental Implants) OR (Dental Prostheses, Surgical) OR (Surgical Dental Prostheses) OR (Surgical Dental Prosthesis) OR (Prosthesis, Surgical Dental)<br><br>#3 “Peri-Implantitis” [MESH] OR (Peri Implantitis) OR (Peri-Implantitides) OR (Periimplantitis) OR (Periimplantitides)<br><br>#4 “Bone Resorption” [MESH] OR (Bone Resorptions) OR (Resorption, Bone) OR (Resorptions, Bone) OR (Osteoclastic Bone Loss) OR (Bone Loss, Osteoclastic) OR (Bone Losses, Osteoclastic) OR (Loss, Osteoclastic Bone) OR (Losses, Osteoclastic Bone) OR (Osteoclastic Bone Losses)<br><br>#5 #1 AND #2<br><br>#6 #1 AND #2 AND #3<br><br>#7 #1 AND #2 AND #4

SCOPUS	#1 “Torque” [MESH] OR (Torques)<br><br>#2 “Dental Implants ” [MESH] OR (Implant, Dental) OR (Implants, Dental) OR (Dental Implants, Mini) OR (Dental Implant, Mini) OR (Mini Dental Implant) OR (Mini Dental Implants) OR (Dental Prostheses, Surgical) OR (Surgical Dental Prostheses) OR (Surgical Dental Prosthesis) OR (Prosthesis, Surgical Dental)<br><br>#3 “Peri-Implantitis” [MESH] OR (Peri Implantitis) OR (Peri-Implantitides) OR (Periimplantitis) OR (Periimplantitides)<br><br>#4 “Bone Resorption” [MESH] OR (Bone Resorptions) OR (Resorption, Bone) OR (Resorptions, Bone) OR (Osteoclastic Bone Loss) OR (Bone Loss, Osteoclastic) OR (Bone Losses, Osteoclastic) OR (Loss, Osteoclastic Bone) OR (Losses, Osteoclastic Bone) OR (Osteoclastic Bone Losses)<br><br>#5 #1 AND #2<br><br>#6 #1 AND #2 AND #3<br><br>#7 #1 AND #2 AND #4

Table S3. Summary table of studies excluded in this systematic review.

Excluded Studies	Exclusion Reasons
Li et al., 2015 [1]	Meta analysis
Roca-Millan et al., 2020 [2]	Systematic review and meta-analysis
Atieh et al., 2021 [3]	Systematic review and meta-analysis
Darriba et al., 2023 [4]	Systematic review and meta-analysis
Berardini et al., 2016 [5]	Systematic review and meta-analysis
Di Stefano et al., 2021 [6]	Narrative review
Ikar et al., 2020 [7]	Systematic review and meta-analysis
Lemos et al., 2020 [8]	Systematic review and meta-analysis
Radi et al., 2023 [9]	Meta analysis

Table S4. Criteria for judging risk of bias in ROBINS-I assessment tool

**1. Reaching risk of bias judgements for bias due to confounding**	
Low risk of bias (the study is comparable to a well-performed randomized trial with regard to this domain)	The investigators describe a random component in the sequence generation process.
Moderate risk of bias (the study is sound for a non-randomized study with regard to this domain but cannot be considered comparable to a well-performed randomized trial)	The investigators describe a non-random component in the sequence generation process. Usually, the description would involve some systematic, non-random approach.<br>Other non-random approaches happen much less frequently than the systematic approaches mentioned above and tend to be obvious. They usually involve judgement or some method of non-random categorization of participants.
Serious risk of bias (the study has some important problems)	
Critical risk of bias (the study is too problematic to provide any useful evidence on the effects of intervention)	Participants and investigators enrolling participants could not foresee assignment because one of the following, or an equivalent method, was used to conceal allocation.
No information on which to base a judgement about risk of bias for this domain	Participants or investigators enrolling participants could possibly foresee assignments and thus introduce selection bias.
**2. Reaching risk of bias judgements for bias in selection of participants into the study**	
Low risk of bias (the study is comparable to a well-performed randomized trial with regard to this domain)	Any one of the following:<br>- No blinding or incomplete blinding, but the review authors judge that the outcome is not likely to be influenced by lack of blinding;<br>- Blinding of participants and key study personnel ensured, and unlikely that the blinding could have been broken;<br>- No blinding of outcome assessment, but the review authors judge that the outcome measurement is not likely to be influenced by lack of blinding;<br>- Blinding of outcome assessment ensured, and unlikely that the blinding could have been broken.
Moderate risk of bias (the study is sound for a non-randomized study with regard to this domain but cannot be considered comparable to a well-performed randomized trial)	Any one of the following:<br>- No blinding or incomplete blinding, and the outcome is likely to be influenced by lack of blinding;

	- Blinding of key study participants and personnel attempted, but likely that the blinding could have been broken, and the outcome is likely to be influenced by lack of blinding;<br>- No blinding of outcome assessment, and the outcome measurement is likely to be influenced by lack of blinding;<br>- Blinding of outcome assessment, but likely that the blinding could have been broken, and the outcome measurement is likely to be influenced by lack of blinding.
Serious risk of bias (the study has some important problems)	
Critical risk of bias (the study is too problematic to provide any useful evidence on the effects of intervention)	Any one of the following:<br>- No missing outcome data;<br>- Reasons for missing outcome data unlikely to be related to true outcome (for survival data, censoring unlikely to be introducing bias);<br>- Missing outcome data balanced in numbers across intervention groups, with similar reasons for missing data across groups;<br>- For dichotomous outcome data, the proportion of missing outcomes compared with observed event risk not enough to have a clinically relevant impact on the intervention effect estimate;<br>- For continuous outcome data, plausible effect size (difference in means or standardized difference in means) among missing outcomes not enough to have a clinically relevant impact on observed effect size;<br>- Missing data have been imputed using appropriate methods.
No information on which to base a judgement about risk of bias for this domain	Any one of the following:<br>- Reason for missing outcome data likely to be related to true outcome, with either imbalance in numbers or reasons for missing data across intervention groups;<br>- For dichotomous outcome data, the proportion of missing outcomes compared with observed event risk enough to induce clinically relevant bias in intervention effect estimate;<br>- For continuous outcome data, plausible effect size (difference in means or standardized difference in means) among missing outcomes enough to induce clinically relevant bias in observed effect size;

	- ‘As-treated’ analysis done with substantial departure of the intervention received from that assigned at randomization;<br>- Potentially inappropriate application of simple imputation.
**3. Reaching risk of bias judgements for bias in classification of interventions**	
Low risk of bias (the study is comparable to a well-performed randomized trial with regard to this domain)	Any one of the following:<br>- The study protocol is available and all of the study’s pre-specified (primary and secondary) outcomes that are of interest in the review have been reported in the pre-specified way;<br>- The study protocol is not available but it is clear that the published reports include all expected outcomes, including those that were pre-specified (convincing text of this nature may be uncommon).
Moderate risk of bias (the study is sound for a non-randomized study with regard to this domain but cannot be considered comparable to a well-performed randomized trial)	Any one of the following:<br>- Not all of the study’s pre-specified primary outcomes have been reported;<br>- One or more primary outcomes is reported using measurements, analysis methods or subsets of the data (e.g., subscales) that were not pre-specified;<br>- One or more reported primary outcomes were not pre-specified (unless clear justification for their reporting is provided, such as an unexpected adverse effect);<br>- One or more outcomes of interest in the review are reported incompletely so that they cannot be entered in a meta-analysis;<br>- The study report fails to include results for a key outcome that would be expected to have been reported for such a study.

Table S5. Risk of bias of the studies included in this review through ROBINS-I assessment tool.

**Authors and Year of Publication**	**Signalling questions**	**Description**	**Response options**
Makary et al., 2011 [10]	**1. Bias due to confounding**		
	1.1 Is there potential for confounding of the effect of intervention in this study?<br>**If N/PN to 1.1:** the study can be considered to be at low risk of bias due to confounding and no further signalling questions need be considered	Preliminary analyses were performed to ensure no violation of the assumption of normality, linearity, and homoscedasticity.	Y / PY / PN / N
	**If Y/PY to 1.1**: determine whether there is a need to assess time-varying confounding:		
	1.2. Was the analysis based on splitting participants' follow up time according to intervention received?<br>**If N/PN**, answer questions relating to baseline confounding (1.4 to 1.6)<br>**If Y/PY**, go to question 1.3.		NA / Y / PY / PN / N / NI
	1.3. Were intervention discontinuations or switches likely to be related to factors that are prognostic for the outcome?<br>**If N/PN**, answer questions relating to baseline confounding (1.4 to 1.6)<br>**If Y/PY**, answer questions relating to both baseline and time-varying confounding (1.7 and 1.8)		NA / Y / PY / PN / N / NI
	Questions relating to baseline confounding only		
	1.4. Did the authors use an appropriate analysis method that controlled for all the important confounding domains?		NA / Y / PY / PN / N / NI
	1.5. **If Y/PY to 1.4**: Were confounding domains that were controlled for measured validly and reliably by the variables available in this study?		NA / Y / PY / PN / N / NI
	1.6. Did the authors control for any post-intervention variables that could have been affected by the intervention?		NA / Y / PY / PN / N / NI
	Questions relating to baseline and time-varying confounding		
	1.7. Did the authors use an appropriate analysis method that controlled for all the important confounding domains and for time-varying confounding?		NA / Y / PY / PN / N / NI
	1.8. **If Y/PY to 1.7**: Were confounding domains that were controlled for measured validly and reliably by the variables available in this study?		NA / Y / PY / PN / N / NI

	Risk of bias judgement		**Low** / Moderate / Serious / Critical / NI
	Optional: What is the predicted direction of bias due to confounding?		Favours experimental / Favours comparator / Unpredictable
	**2. Bias in selection of participants into the study**		
	2.1. Was selection of participants into the study (or into the analysis) based on participant characteristics observed after the start of intervention?<br>**If N/PN to 2.1:** go to 2.4	All the patients were in good health, with no systemic disorders. All were accurately informed about the procedures, and all signed an informed consent form.	Y / PY / PN / N / NI
	2.2. **If Y/PY to 2.1**: Were the post-intervention variables that influenced selection likely to be associated with intervention?<br>2.3 **If Y/PY to 2.2**: Were the post-intervention variables that influenced selection likely to be influenced by the outcome or a cause of the outcome?		NA / Y / PY / PN /N / NI<br><br>NA / Y / PY / PN / N / NI
	2.4. Do start of follow-up and start of intervention coincide for most participants?		Y/ PY / PN / N / **NI**
	2.5. **If Y/PY to 2.2 and 2.3, or N/PN to 2.4**: Were adjustment techniques used that are likely to correct for the presence of selection biases?		NA / Y / PY / PN / N / NI
	Risk of bias judgement		**Low** / Moderate / Serious / Critical / NI

	Optional: What is the predicted direction of bias due to selection of participants into the study?		**Favours experimental** / Favours comparator / Towards null /Away from null / Unpredictable
	**3. Bias in classification of interventions**		
	3.1 Were intervention groups clearly defined?		**Y** / PY / PN / N / NI
	3.2 Was the information used to define intervention groups recorded at the start of the intervention?		Y/ PY / PN / N / NI
	3.3 Could classification of intervention status have been affected by knowledge of the outcome or risk of the outcome?		Y / PY / PN / **N** / NI
	Risk of bias judgement		**Low** / Moderate / Serious / Critical / NI
	Optional: What is the predicted direction of bias due to classification of interventions?		Favours experimental / Favours comparator / Towards null /Away from null / Unpredictable
	**4. Bias due to deviations from intended interventions**		
	If your aim for this study is to assess the effect of assignment to intervention, answer questions 4.1 and 4.2		
	4.1. Were there deviations from the intended intervention beyond what would be expected in usual practice?	All interventions were performed according to clinical practice.	Y / PY / PN / **N** / NI

	4.2. **If Y/PY to 4.1**: Were these deviations from intended intervention unbalanced between groups *and* likely to have affected the outcome?		NA / Y / PY / PN / N / NI
	If your aim for this study is to assess the effect of starting and adhering to intervention, answer questions 4.3 to 4.6		
	4.3. Were important co-interventions balanced across intervention groups?		Y / PY / PN / N / NI
	4.4. Was the intervention implemented successfully for most participants?		Y / PY / PN / N / NI
	4.5. Did study participants adhere to the assigned intervention regimen?		Y / PY / PN / N / NI
	4.6. **If N/PN to 4.3, 4.4 or 4.5**: Was an appropriate analysis used to estimate the effect of starting and adhering to the intervention?		NA / Y / PY / PN / N / NI
	Risk of bias judgement		**Low** / Moderate / Serious / Critical / NI
	Optional: What is the predicted direction of bias due to deviations from the intended interventions?		**Favours experimental** / Favours comparator / Towards null /Away from null / Unpredictable
	**5. Bias due to missing data**		
	5.1 Were outcome data available for all, or nearly all, participants?		Y / PY / PN / N / NI
	5.2 Were participants excluded due to missing data on intervention status?		Y / PY / PN / N / NI
	5.3 Were participants excluded due to missing data on other variables needed for the analysis?		Y / PY / PN / N / NI
	5.4 **If PN/N to 5.1, or Y/PY to 5.2 or 5.3**: Are the proportion of participants and reasons for missing data similar across interventions?		NA / Y / PY / PN / N / NI
	5.5 **If PN/N to 5.1, or Y/PY to 5.2 or 5.3**: Is there evidence that results were robust to the presence of missing data?		NA / Y / PY / PN / N / NI

	Risk of bias judgement		**Low** / Moderate / Serious / Critical / NI
	Optional: What is the predicted direction of bias due to missing data?		**Favours experimental** / Favours comparator / Towards null /Away from null / Unpredictable
	**6. Bias in measurement of outcomes**		
	6.1 Could the outcome measure have been influenced by knowledge of the intervention received?		Y / PY / PN / N / NI
	6.2 Were outcome assessors aware of the intervention received by study participants?		Y / PY / PN / N / NI
	6.3 Were the methods of outcome assessment comparable across intervention groups?		Y / PY / PN / N / NI
	6.4 Were any systematic errors in measurement of the outcome related to intervention received?		Y / PY / PN / N / NI
	Risk of bias judgement		**Low** / Moderate / Serious / Critical / NI
	Optional: What is the predicted direction of bias due to measurement of outcomes?		**Favours experimental** / Favours comparator / Towards null /Away from null / Unpredictable

	**7. Bias in selection of the reported result**		
	Is the reported effect estimate likely to be selected, on the basis of the results, from...		
	7.1. ... multiple outcome *measurements* within the outcome domain?		Y / PY / PN / N / NI
	7.2 ... multiple *analyses* of the intervention-outcome relationship?		Y / PY / PN / N / NI
	7.3 ... different *subgroups*?		Y / PY / PN / N / NI
	Risk of bias judgement		**Low** / Moderate / Serious / Critical / NI
	Optional: What is the predicted direction of bias due to selection of the reported result?		**Favours experimental** / Favours comparator / Towards null /Away from null / Unpredictable
	**Overall bias**		
	Risk of bias judgement		**Low** / Moderate / Serious / Critical / NI

	Optional: What is the overall predicted direction of bias for this outcome?		**Favours experimental** / Favours comparator / Towards null /Away from null / Unpredictable
Oskouei et al, 2023 [11]	**1. Bias due to confounding**		
	1.1 Is there potential for confounding of the effect of intervention in this study?<br>**If** N/PN **to 1.1:** the study can be considered to be at low risk of bias due to confounding and no further signalling questions need be considered	To ensure the maximum scienti c accuracy of the results,<br>all stages of the research were conducted carefully based on the principles of scientific research	Y / PY / PN / N
	**If** Y/PY **to 1.1**: determine whether there is a need to assess time-varying confounding:		
	1.2. Was the analysis based on splitting participants' follow up time according to intervention received?<br>**If N/PN**, answer questions relating to baseline confounding (1.4 to 1.6)<br>**If Y/PY**, go to question 1.3.		NA / Y / PY / PN / N / NI
	1.3. Were intervention discontinuations or switches likely to be related to factors that are prognostic for the outcome?<br>**If N/PN**, answer questions relating to baseline confounding (1.4 to 1.6)<br>**If Y/PY**, answer questions relating to both baseline and time-varying confounding (1.7 and 1.8)		NA / Y / PY / PN / N / NI
	Questions relating to baseline confounding only		
	1.4. Did the authors use an appropriate analysis method that controlled for all the important confounding domains?		NA / Y / PY / PN / N / NI
	1.5. **If** Y/PY **to 1.4**: Were confounding domains that were controlled for measured validly and reliably by the variables available in this study?		NA / Y / PY / PN / N / NI
	1.6. Did the authors control for any post-intervention variables that could have been affected by the intervention?		NA / Y / PY / PN / N / NI
	Questions relating to baseline and time-varying confounding		

	1.7. Did the authors use an appropriate analysis method that controlled for all the important confounding domains and for time-varying confounding?		NA / Y / PY / PN / N / NI
	1.8. **If** Y/PY **to 1.7**: Were confounding domains that were controlled for measured validly and reliably by the variables available in this study?		NA / Y / PY / PN / N / NI
	Risk of bias judgement		**Low** / Moderate / Serious / Critical / NI
	Optional: What is the predicted direction of bias due to confounding?		**Favours experimental** / Favours comparator / Unpredictable
	**2. Bias in selection of participants into the study**		
	2.1. Was selection of participants into the study (or into the analysis) based on participant characteristics observed after the start of intervention?<br>**If** N/PN **to 2.1:** go to 2.4	Selection of participants took place after start of intervention, as it was a retrospective study.	Y / PY / PN / N / NI
	2.2. **If** Y/PY **to 2.1**: Were the post-intervention variables that influenced selection likely to be associated with intervention?<br>2.3 **If** Y/PY **to 2.2**: Were the post-intervention variables that influenced selection likely to be influenced by the outcome or a cause of the outcome?		NA / Y / PY / PN / N / NI<br><br>NA / Y / PY / PN / N / NI
	2.4. Do start of follow-up and start of intervention coincide for most participants?		Y / PY / PN / N / **NI**
	2.5. **If** Y/PY **to 2.2 and 2.3, or** N/PN **to 2.4**: Were adjustment techniques used that are likely to correct for the presence of selection biases?		NA / Y / PY / PN / N / NI
	Risk of bias judgement		**Low** / Moderate / Serious / Critical / NI

	Optional: What is the predicted direction of bias due to selection of participants into the study?		**Favours experimental** / Favours comparator / Towards null /Away from null / Unpredictable
	**3. Bias in classification of interventions**		
	3.1 Were intervention groups clearly defined?		Y / PY / PN / N / NI
	3.2 Was the information used to define intervention groups recorded at the start of the intervention?		Y / PY / PN / N / NI
	3.3 Could classification of intervention status have been affected by knowledge of the outcome or risk of the outcome?		Y / PY / PN / N / NI
	Risk of bias judgement		**Low** / Moderate / Serious / Critical / NI
	Optional: What is the predicted direction of bias due to classification of interventions?		**Favours experimental** / Favours comparator / Towards null /Away from null / Unpredictable
	**4. Bias due to deviations from intended interventions**		
	If your aim for this study is to assess the effect of assignment to intervention, answer questions 4.1 and 4.2		
	4.1. Were there deviations from the intended intervention beyond what would be expected in usual practice?	All interventions were performed according to clinical practice.	Y / PY / PN / N / NI
	4.2. **If Y/PY to 4.1**: Were these deviations from intended intervention unbalanced between groups *and* likely to have affected the outcome?		NA / Y / PY / PN / N / NI
	If your aim for this study is to assess the effect of starting and adhering to intervention, answer questions 4.3 to 4.6		

	4.3. Were important co-interventions balanced across intervention groups?		Y / PY / PN / N / NI
	4.4. Was the intervention implemented successfully for most participants?		Y / PY / PN / N / NI
	4.5. Did study participants adhere to the assigned intervention regimen?		Y / PY / PN / N / NI
	4.6. **If N/PN to 4.3, 4.4 or 4.5**: Was an appropriate analysis used to estimate the effect of starting and adhering to the intervention?		NA / Y / PY / PN / N / NI
	Risk of bias judgement		**Low** / Moderate / Serious / Critical / NI
	Optional: What is the predicted direction of bias due to deviations from the intended interventions?		**Favours experimental** / Favours comparator / Towards null /Away from null / Unpredictable
	**5. Bias due to missing data**		
	5.1 Were outcome data available for all, or nearly all, participants?		Y / PY / PN / N / NI
	5.2 Were participants excluded due to missing data on intervention status?		Y / PY / PN /N/ NI
	5.3 Were participants excluded due to missing data on other variables needed for the analysis?		Y / PY / PN / N / NI
	5.4 **If PN/N to 5.1, or Y/PY to 5.2 or 5.3**: Are the proportion of participants and reasons for missing data similar across interventions?		NA / Y / PY / PN / N / **NI**
	5.5 **If PN/N to 5.1, or Y/PY to 5.2 or 5.3**: Is there evidence that results were robust to the presence of missing data?		NA / Y / PY / PN / N / NI
	Risk of bias judgement		Low / **Moderate** / Serious / Critical / NI

	Optional: What is the predicted direction of bias due to missing data?		**<u>Favours experimental</u>** / Favours comparator / Towards null /Away from null / Unpredictable
	**6. Bias in measurement of outcomes**		
	6.1 Could the outcome measure have been influenced by knowledge of the intervention received?		Y / PY / PN / <u>N</u> / NI
	6.2 Were outcome assessors aware of the intervention received by study participants?		Y / PY / PN / N / NI
	6.3 Were the methods of outcome assessment comparable across intervention groups?		<u>Y</u> / PY / PN / N / NI
	6.4 Were any systematic errors in measurement of the outcome related to intervention received?		Y / PY / PN / <u>N</u> / NI
	Risk of bias judgement		**<u>Low</u>** / Moderate / Serious / Critical / NI
	Optional: What is the predicted direction of bias due to measurement of outcomes?		**<u>Favours experimental</u>** / Favours comparator / Towards null /Away from null / Unpredictable
	**7. Bias in selection of the reported result**		
	Is the reported effect estimate likely to be selected, on the basis of the results, from...		
	7.1. ... multiple outcome *measurements* within the outcome domain?		Y / PY / PN / <u>N</u> / NI

	7.2 ... multiple *analyses* of the intervention-outcome relationship?		Y / PY / PN / N / NI
	7.3 ... different *subgroups*?		Y / PY / PN / N / NI
	Risk of bias judgement		**Low** / Moderate / Serious / Critical / NI
	Optional: What is the predicted direction of bias due to selection of the reported result?		**Favours experimental** / Favours comparator / Towards null /Away from null / Unpredictable
	**Overall bias**		
	Risk of bias judgement		**Low** / Moderate / Serious / Critical / NI
	Optional: What is the overall predicted direction of bias for this outcome?		**Favours experimental** / Favours comparator / Towards null /Away from null / Unpredictable
Bidgoli et al., 2015 [12]	**1. Bias due to confounding**		
	1.1 Is there potential for confounding of the effect of intervention in this study?<br>**If N/PN to 1.1:** the study can be considered to be at low risk of bias due to confounding and no further signalling questions need be considered	Patients with fractured dental implants due to trauma were excluded.	Y / PY / PN / N
	**If Y/PY to 1.1**: determine whether there is a need to assess time-varying confounding:		

	1.2. Was the analysis based on splitting participants' follow up time according to intervention received?<br>**If N/PN**, answer questions relating to baseline confounding (1.4 to 1.6)<br>**If Y/PY**, go to question 1.3.<br>1.3. Were intervention discontinuations or switches likely to be related to factors that are prognostic for the outcome?<br>**If N/PN**, answer questions relating to baseline confounding (1.4 to 1.6)<br>**If Y/PY**, answer questions relating to both baseline and time-varying confounding (1.7 and 1.8)		NA / Y / PY / PN / N / NI<br><br><br>NA / Y / PY / PN / N / NI
	Questions relating to baseline confounding only		
	1.4. Did the authors use an appropriate analysis method that controlled for all the important confounding domains?		NA / Y / PY / PN / N / NI
	1.5. **If** Y/PY **to 1.4**: Were confounding domains that were controlled for measured validly and reliably by the variables available in this study?		NA / Y / PY / PN / N / NI
	1.6. Did the authors control for any post-intervention variables that could have been affected by the intervention?		NA / Y / PY / PN / N / NI
	Questions relating to baseline and time-varying confounding		
	1.7. Did the authors use an appropriate analysis method that controlled for all the important confounding domains and for time-varying confounding?		NA / Y / PY / PN / N / NI
	1.8. **If** Y/PY **to 1.7**: Were confounding domains that were controlled for measured validly and reliably by the variables available in this study?		NA / Y / PY / PN / N / NI
	Risk of bias judgement		**Low** / Moderate / Serious / Critical / NI
	Optional: What is the predicted direction of bias due to confounding?		**Favours experimental** / Favours comparator / Unpredictable
	**2. Bias in selection of participants into the study**		
	2.1. Was selection of participants into the study (or into the analysis) based on participant characteristics observed after the start of intervention?<br>**If** N/PN **to 2.1:** go to 2.4	Selection of participants took place after start of intervention, as it was a retrospective study.	Y / PY / PN / N / NI

	2.2. **If Y/PY to 2.1**: Were the post-intervention variables that influenced selection likely to be associated with intervention?<br>2.3 **If Y/PY to 2.2**: Were the post-intervention variables that influenced selection likely to be influenced by the outcome or a cause of the outcome?		NA / Y / PY / PN / N / NI<br><br>NA / Y / PY / PN / N / NI
	2.4. Do start of follow-up and start of intervention coincide for most participants?		Y / PY / PN / N / **NI**
	2.5. **If Y/PY to 2.2 and 2.3, or N/PN to 2.4**: Were adjustment techniques used that are likely to correct for the presence of selection biases?		NA / Y / PY / PN / N / NI
	Risk of bias judgement		**Low** / Moderate / Serious / Critical / NI
	Optional: What is the predicted direction of bias due to selection of participants into the study?		**Favours experimental** / Favours comparator / Towards null /Away from null / Unpredictable
	**3. Bias in classification of interventions**		
	3.1 Were intervention groups clearly defined?	High torque group (45-70Ncm) and low torque group (20-30Ncm).	Y / PY / PN / N / NI
	3.2 Was the information used to define intervention groups recorded at the start of the intervention?		Y / PY / PN / N / NI
	3.3 Could classification of intervention status have been affected by knowledge of the outcome or risk of the outcome?		Y / PY / PN / N / NI
	Risk of bias judgement		**Low** / Moderate / Serious / Critical / NI

	Optional: What is the predicted direction of bias due to classification of interventions?		**Favours experimental** / Favours comparator / Towards null /Away from null / Unpredictable
	**4. Bias due to deviations from intended interventions**		
	If your aim for this study is to assess the effect of assignment to intervention, answer questions 4.1 and 4.2		
	4.1. Were there deviations from the intended intervention beyond what would be expected in usual practice?	All interventions were performed according to clinical practice.	Y / PY / PN / N / NI
	4.2. **If Y/PY to 4.1**: Were these deviations from intended intervention unbalanced between groups *and* likely to have affected the outcome?		NA / Y / PY / PN / N / NI
	If your aim for this study is to assess the effect of starting and adhering to intervention, answer questions 4.3 to 4.6		
	4.3. Were important co-interventions balanced across intervention groups?		Y / PY / PN / N / NI
	4.4. Was the intervention implemented successfully for most participants?		Y / PY / PN / N / NI
	4.5. Did study participants adhere to the assigned intervention regimen?		Y / PY / PN / N / NI
	4.6. **If N/PN to 4.3, 4.4 or 4.5**: Was an appropriate analysis used to estimate the effect of starting and adhering to the intervention?		NA / Y / PY / PN / N / NI
	Risk of bias judgement		**Low** / Moderate / Serious / Critical / NI

	Optional: What is the predicted direction of bias due to deviations from the intended interventions?		**Favours experimental** / Favours comparator / Towards null /Away from null / Unpredictable
	**5. Bias due to missing data**		
	5.1 Were outcome data available for all, or nearly all, participants?		Y / PY / PN / N / NI
	5.2 Were participants excluded due to missing data on intervention status?		Y / PY / PN / N / NI
	5.3 Were participants excluded due to missing data on other variables needed for the analysis?		Y / PY / PN / N / NI
	5.4 **If PN/N to 5.1, or Y/PY to 5.2 or 5.3**: Are the proportion of participants and reasons for missing data similar across interventions?		NA / Y / PY / PN / N / NI
	5.5 **If PN/N to 5.1, or Y/PY to 5.2 or 5.3**: Is there evidence that results were robust to the presence of missing data?		NA / Y / PY / PN / N / NI
	Risk of bias judgement		**Low** / Moderate / Serious / Critical / NI
	Optional: What is the predicted direction of bias due to missing data?		**Favours experimental** / Favours comparator / Towards null /Away from null / Unpredictable
	**6. Bias in measurement of outcomes**		
	6.1 Could the outcome measure have been influenced by knowledge of the intervention received?		Y / PY / PN / N / NI

	6.2 Were outcome assessors aware of the intervention received by study participants?		Y / PY / PN / N / NI
	6.3 Were the methods of outcome assessment comparable across intervention groups?		Y / PY / PN / N / NI
	6.4 Were any systematic errors in measurement of the outcome related to intervention received?		Y / PY / PN / N / NI
	Risk of bias judgement		**Low** / Moderate / Serious / Critical / NI
	Optional: What is the predicted direction of bias due to measurement of outcomes?		**Favours experimental** / Favours comparator / Towards null /Away from null / Unpredictable
	**7. Bias in selection of the reported result**		
	Is the reported effect estimate likely to be selected, on the basis of the results, from...		
	7.1. ... multiple outcome *measurements* within the outcome domain?		Y / PY / PN / N / NI
	7.2 ... multiple *analyses* of the intervention-outcome relationship?		Y / PY / PN / N / NI
	7.3 ... different *subgroups*?		Y / PY / PN / N / NI
	Risk of bias judgement		**Low** / Moderate / Serious / Critical / NI

	Optional: What is the predicted direction of bias due to selection of the reported result?		**Favours experimental** / Favours comparator / Towards null /Away from null / Unpredictable
	**Overall bias**		
	Risk of bias judgement		**Low** / Moderate / Serious / Critical / NI
	Optional: What is the overall predicted direction of bias for this outcome?		**Favours experimental** / Favours comparator / Towards null /Away from null / Unpredictable
Grandi et al., 2012 [13]	**1. Bias due to confounding**		
	1.1 Is there potential for confounding of the effect of intervention in this study?<br>**If N/PN to 1.1:** the study can be considered to be at low risk of bias due to confounding and no further signalling questions need be considered		Y / PY / PN / N
	**If Y/PY to 1.1**: determine whether there is a need to assess time-varying confounding:		
	1.2. Was the analysis based on splitting participants' follow up time according to intervention received?<br>**If N/PN**, answer questions relating to baseline confounding (1.4 to 1.6)<br>**If Y/PY**, go to question 1.3.		NA / Y / PY / PN / N / NI
	1.3. Were intervention discontinuations or switches likely to be related to factors that are prognostic for the outcome?<br>**If N/PN**, answer questions relating to baseline confounding (1.4 to 1.6)<br>**If Y/PY**, answer questions relating to both baseline and time-varying confounding (1.7 and 1.8)		NA / Y / PY / PN / N / NI

	Questions relating to baseline confounding only		
	1.4. Did the authors use an appropriate analysis method that controlled for all the important confounding domains?		NA / Y / PY / PN / N / NI
	1.5. **If** Y/PY **to 1.4**: Were confounding domains that were controlled for measured validly and reliably by the variables available in this study?		NA / Y / PY / PN / N / NI
	1.6. Did the authors control for any post-intervention variables that could have been affected by the intervention?		NA / Y / PY / PN / N / NI
	Questions relating to baseline and time-varying confounding		
	1.7. Did the authors use an appropriate analysis method that controlled for all the important confounding domains and for time-varying confounding?		NA / Y / PY / PN / N / NI
	1.8. **If** Y/PY **to 1.7**: Were confounding domains that were controlled for measured validly and reliably by the variables available in this study?		NA / Y / PY / PN / N / NI
	Risk of bias judgement		**Low** / Moderate / Serious / Critical / NI
	Optional: What is the predicted direction of bias due to confounding?		**Favours experimental** / Favours comparator / Unpredictable
	**2. Bias in selection of participants into the study**		
	2.1. Was selection of participants into the study (or into the analysis) based on participant characteristics observed after the start of intervention?<br>**If** N/PN **to 2.1:** go to 2.4		Y / PY / PN / N / NI
	2.2. **If** Y/PY **to 2.1**: Were the post-intervention variables that influenced selection likely to be associated with intervention?<br>2.3 **If** Y/PY **to 2.2**: Were the post-intervention variables that influenced selection likely to be influenced by the outcome or a cause of the outcome?		NA / Y / PY / PN / N / NI<br>NA / Y / PY / PN / N / NI
	2.4. Do start of follow-up and start of intervention coincide for most participants?		Y / PY / PN / N / NI
	2.5. **If** Y/PY **to 2.2 and 2.3, or** N/PN **to 2.4**: Were adjustment techniques used that are likely to correct for the presence of selection biases?		NA / Y / PY / PN / N / NI

	Risk of bias judgement		**Low** / Moderate / Serious / Critical / NI
	Optional: What is the predicted direction of bias due to selection of participants into the study?		**Favours experimental** / Favours comparator / Towards null /Away from null / Unpredictable
	**3. Bias in classification of interventions**		
	3.1 Were intervention groups clearly defined?	High torque group (50-80Ncm), regular torque group (30-45Ncm)	Y / PY / PN / N / NI
	3.2 Was the information used to define intervention groups recorded at the start of the intervention?		Y / PY / PN / N / NI
	3.3 Could classification of intervention status have been affected by knowledge of the outcome or risk of the outcome?		Y / PY / PN / N / NI
	Risk of bias judgement		**Low** / Moderate / Serious / Critical / NI
	Optional: What is the predicted direction of bias due to classification of interventions?		**Favours experimental** / Favours comparator / Towards null /Away from null / Unpredictable

	**4. Bias due to deviations from intended interventions**		
	If your aim for this study is to assess the effect of assignment to intervention, answer questions 4.1 and 4.2		
	4.1. Were there deviations from the intended intervention beyond what would be expected in usual practice?	All interventions were performed according to clinical practice.	Y / PY / PN / N / NI
	4.2. **If Y/PY to 4.1**: Were these deviations from intended intervention unbalanced between groups *and* likely to have affected the outcome?		NA / Y / PY / PN / N / NI
	If your aim for this study is to assess the effect of starting and adhering to intervention, answer questions 4.3 to 4.6		
	4.3. Were important co-interventions balanced across intervention groups?		Y / PY / PN / N / NI
	4.4. Was the intervention implemented successfully for most participants?		Y / PY / PN / N / NI
	4.5. Did study participants adhere to the assigned intervention regimen?		Y / PY / PN / N / NI
	4.6. **If N/PN to 4.3, 4.4 or 4.5**: Was an appropriate analysis used to estimate the effect of starting and adhering to the intervention?		NA / Y / PY / PN / N / NI
	Risk of bias judgement		**Low** / Moderate / Serious / Critical / NI
	Optional: What is the predicted direction of bias due to deviations from the intended interventions?		**Favours experimental** / Favours comparator / Towards null /Away from null / Unpredictable
	**5. Bias due to missing data**		
	5.1 Were outcome data available for all, or nearly all, participants?		Y / PY / PN / N / NI
	5.2 Were participants excluded due to missing data on intervention status?		Y / PY / PN / N / NI
	5.3 Were participants excluded due to missing data on other variables needed for the analysis?		Y / PY / PN / N / NI

	5.4 **If PN/N to 5.1, or Y/PY to 5.2 or 5.3**: Are the proportion of participants and reasons for missing data similar across interventions?		NA / Y / PY / PN / N / NI
	5.5 **If PN/N to 5.1, or Y/PY to 5.2 or 5.3**: Is there evidence that results were robust to the presence of missing data?		NA / Y / PY / PN / N / NI
	Risk of bias judgement		**Low** / Moderate / Serious / Critical / NI
	Optional: What is the predicted direction of bias due to missing data?		**Favours experimental** / Favours comparator / Towards null /Away from null / Unpredictable
	**6. Bias in measurement of outcomes**		
	6.1 Could the outcome measure have been influenced by knowledge of the intervention received?		Y / PY / PN / N / NI
	6.2 Were outcome assessors aware of the intervention received by study participants?		Y / PY / PN / N / NI
	6.3 Were the methods of outcome assessment comparable across intervention groups?		Y / PY / PN / N / NI
	6.4 Were any systematic errors in measurement of the outcome related to intervention received?		Y / PY / PN / N / NI
	Risk of bias judgement		**Low** / Moderate / Serious / Critical / NI

	Optional: What is the predicted direction of bias due to measurement of outcomes?		**Favours experimental** / Favours comparator / Towards null /Away from null / Unpredictable
	**7. Bias in selection of the reported result**		
	Is the reported effect estimate likely to be selected, on the basis of the results, from...		
	7.1. ... multiple outcome *measurements* within the outcome domain?		Y / PY / PN / N / NI
	7.2 ... multiple *analyses* of the intervention-outcome relationship?		Y / PY / PN / N / NI
	7.3 ... different *subgroups*?		Y / PY / PN / N / NI
	Risk of bias judgement		**Low** / Moderate / Serious / Critical / NI
	Optional: What is the predicted direction of bias due to selection of the reported result?		**Favours experimental** / Favours comparator / Towards null /Away from null / Unpredictable
	**Overall bias**		
	Risk of bias judgement		**Low** / Moderate / Serious / Critical / NI

	Optional: What is the overall predicted direction of bias for this outcome?		**Favours experimental** / Favours comparator / Towards null /Away from null / Unpredictable
Rizkallah et al., 2013 [14]	**1. Bias due to confounding**		
	1.1 Is there potential for confounding of the effect of intervention in this study?<br>**If N/PN to 1.1:** the study can be considered to be at low risk of bias due to confounding and no further signalling questions need be considered	All of the cases were performed by the same attending surgeon or by sur- gical residents under direct supervision of that attending surgeon.	Y / PY / PN / N
	**If Y/PY to 1.1**: determine whether there is a need to assess time-varying confounding:		
	1.2. Was the analysis based on splitting participants' follow up time according to intervention received?<br>**If N/PN**, answer questions relating to baseline confounding (1.4 to 1.6)<br>**If Y/PY**, go to question 1.3.<br>1.3. Were intervention discontinuations or switches likely to be related to factors that are prognostic for the outcome?<br>**If N/PN**, answer questions relating to baseline confounding (1.4 to 1.6)<br>**If Y/PY**, answer questions relating to both baseline and time-varying confounding (1.7 and 1.8)		NA / Y / PY / PN / N / NI<br><br>NA / Y / PY / PN / N / NI
	Questions relating to baseline confounding only		
	1.4. Did the authors use an appropriate analysis method that controlled for all the important confounding domains?		NA / Y / PY / PN / N / NI
	1.5. **If Y/PY to 1.4**: Were confounding domains that were controlled for measured validly and reliably by the variables available in this study?		NA / Y / PY / PN / N / NI
	1.6. Did the authors control for any post-intervention variables that could have been affected by the intervention?		NA / Y / PY / PN / N / NI
	Questions relating to baseline and time-varying confounding		
	1.7. Did the authors use an appropriate analysis method that controlled for all the important confounding domains and for time-varying confounding?		NA / Y / PY / PN / N / NI

	1.8. **If** Y/PY **to 1.7**: Were confounding domains that were controlled for measured validly and reliably by the variables available in this study?		NA / Y / PY / PN / N / NI
	Risk of bias judgement		**Low** / Moderate / Serious / Critical / NI
	Optional: What is the predicted direction of bias due to confounding?		**Favours experimental** / Favours comparator / Unpredictable
	**2. Bias in selection of participants into the study**		
	2.1. Was selection of participants into the study (or into the analysis) based on participant characteristics observed after the start of intervention?<br>**If** N/PN **to 2.1:** go to 2.4	Selection of participants took place after start of intervention, as it was a retrospective study.	Y / PY / PN / N / NI
	2.2. **If** Y/PY **to 2.1**: Were the post-intervention variables that influenced selection likely to be associated with intervention?<br>2.3 **If** Y/PY **to 2.2**: Were the post-intervention variables that influenced selection likely to be influenced by the outcome or a cause of the outcome?		NA / Y / PY / PN / N / NI<br><br>NA / Y / PY / PN / N / NI
	2.4. Do start of follow-up and start of intervention coincide for most participants?		Y / PY / PN / N / **NI**
	2.5. **If** Y/PY **to 2.2 and 2.3, or** N/PN **to 2.4**: Were adjustment techniques used that are likely to correct for the presence of selection biases?		NA / Y / PY / PN / N / NI
	Risk of bias judgement		**Low** / Moderate / Serious / Critical / NI

	Optional: What is the predicted direction of bias due to selection of participants into the study?		**Favours experimental** / Favours comparator / Towards null /Away from null / Unpredictable
	**3. Bias in classification of interventions**		
	3.1 Were intervention groups clearly defined?		Y/ PY / PN / N / NI
	3.2 Was the information used to define intervention groups recorded at the start of the intervention?		Y / PY / PN / N / NI
	3.3 Could classification of intervention status have been affected by knowledge of the outcome or risk of the outcome?		Y / PY / PN / N / NI
	Risk of bias judgement		**Low** / Moderate / Serious / Critical / NI
	Optional: What is the predicted direction of bias due to classification of interventions?		**Favours experimental** / Favours comparator / Towards null /Away from null / Unpredictable
	**4. Bias due to deviations from intended interventions**		
	If your aim for this study is to assess the effect of assignment to intervention, answer questions 4.1 and 4.2		
	4.1. Were there deviations from the intended intervention beyond what would be expected in usual practice?	All interventions were performed according to clinical practice.	Y / PY / PN / N / NI
	4.2. **If Y/PY to 4.1**: Were these deviations from intended intervention unbalanced between groups *and* likely to have affected the outcome?		NA / Y / PY / PN / N / NI
	If your aim for this study is to assess the effect of starting and adhering to intervention, answer questions 4.3 to 4.6		

	4.3. Were important co-interventions balanced across intervention groups?		Y / PY / PN / N / NI
	4.4. Was the intervention implemented successfully for most participants?		Y / PY / PN / N / NI
	4.5. Did study participants adhere to the assigned intervention regimen?		Y / PY / PN / N / NI
	4.6. **If N/PN to 4.3, 4.4 or 4.5**: Was an appropriate analysis used to estimate the effect of starting and adhering to the intervention?		NA / Y / PY / PN / N / NI
	Risk of bias judgement		**Low** / Moderate / Serious / Critical / NI
	Optional: What is the predicted direction of bias due to deviations from the intended interventions?		**Favours experimental** / Favours comparator / Towards null /Away from null / Unpredictable
	**5. Bias due to missing data**		
	5.1 Were outcome data available for all, or nearly all, participants?		Y / PY / PN / N / NI
	5.2 Were participants excluded due to missing data on intervention status?		Y / PY / PN / N / NI
	5.3 Were participants excluded due to missing data on other variables needed for the analysis?		Y / PY / PN / N / NI
	5.4 **If PN/N to 5.1, or Y/PY to 5.2 or 5.3**: Are the proportion of participants and reasons for missing data similar across interventions?		NA / Y / PY / PN / N / NI
	5.5 **If PN/N to 5.1, or Y/PY to 5.2 or 5.3**: Is there evidence that results were robust to the presence of missing data?		NA / Y / PY / PN / N / NI
	Risk of bias judgement		**Low** / Moderate / Serious / Critical / NI

	Optional: What is the predicted direction of bias due to missing data?		**Favours experimental** / Favours comparator / Towards null /Away from null / Unpredictable
	**6. Bias in measurement of outcomes**		
	6.1 Could the outcome measure have been influenced by knowledge of the intervention received?		Y / PY / PN / N / NI
	6.2 Were outcome assessors aware of the intervention received by study participants?	Retrospective study.	Y / PY / PN / N / NI
	6.3 Were the methods of outcome assessment comparable across intervention groups?		Y / PY / PN / N / NI
	6.4 Were any systematic errors in measurement of the outcome related to intervention received?		Y / PY / PN / N / NI
	Risk of bias judgement		**Low** / Moderate / Serious / Critical / NI
	Optional: What is the predicted direction of bias due to measurement of outcomes?		**Favours experimental** / Favours comparator / Towards null /Away from null / Unpredictable
	**7. Bias in selection of the reported result**		
	Is the reported effect estimate likely to be selected, on the basis of the results, from...		
	7.1. ... multiple outcome *measurements* within the outcome domain?		Y / PY / PN / N / NI

	7.2 ... multiple *analyses* of the intervention-outcome relationship?		Y / PY / PN / N / NI
	7.3 ... different *subgroups*?		Y / PY / PN / N / NI
	Risk of bias judgement		**Low** / Moderate / Serious / Critical / NI
	Optional: What is the predicted direction of bias due to selection of the reported result?		**Favours experimental** / Favours comparator / Towards null /Away from null / Unpredictable
	**Overall bias**		
	Risk of bias judgement		**Low** / Moderate / Serious / Critical / NI
	Optional: What is the overall predicted direction of bias for this outcome?		**Favours experimental** / Favours comparator / Towards null /Away from null / Unpredictable

Khayat et al., 2011 [15]	**1. Bias due to confounding**		
	1.1 Is there potential for confounding of the effect of intervention in this study?<br>**If N/PN to 1.1:** the study can be considered to be at low risk of bias due to confounding and no further signalling questions need be considered	All patients were subjected to a preliminary evaluation that included careful review of their medical and dental histories, detailed clinical examination, and evaluation of oral hygiene.	Y / PY / PN / N
	**If Y/PY to 1.1**: determine whether there is a need to assess time-varying confounding:		
	1.2. Was the analysis based on splitting participants' follow up time according to intervention received?<br>**If N/PN**, answer questions relating to baseline confounding (1.4 to 1.6)<br>**If Y/PY**, go to question 1.3.<br>1.3. Were intervention discontinuations or switches likely to be related to factors that are prognostic for the outcome?<br>**If N/PN**, answer questions relating to baseline confounding (1.4 to 1.6)<br>**If Y/PY**, answer questions relating to both baseline and time-varying confounding (1.7 and 1.8)		NA / Y / PY / PN / N / NI<br>NA / Y / PY / PN / N / NI
	Questions relating to baseline confounding only		
	1.4. Did the authors use an appropriate analysis method that controlled for all the important confounding domains?		NA / Y / PY / PN / N / NI
	1.5. **If Y/PY to 1.4**: Were confounding domains that were controlled for measured validly and reliably by the variables available in this study?		NA / Y / PY / PN / N / NI
	1.6. Did the authors control for any post-intervention variables that could have been affected by the intervention?		NA / Y / PY / PN / N / NI
	Questions relating to baseline and time-varying confounding		
	1.7. Did the authors use an appropriate analysis method that controlled for all the important confounding domains and for time-varying confounding?		NA / Y / PY / PN / N / NI
	1.8. **If Y/PY to 1.7**: Were confounding domains that were controlled for measured validly and reliably by the variables available in this study?		NA / Y / PY / PN / N / NI
	Risk of bias judgement		**Low** / Moderate / Serious / Critical / NI

	Optional: What is the predicted direction of bias due to confounding?		**Favours experimental** / Favours comparator / Unpredictable
	**2. Bias in selection of participants into the study**		
	2.1. Was selection of participants into the study (or into the analysis) based on participant characteristics observed after the start of intervention?<br>**If N/PN to 2.1:** go to 2.4		Y / PY / PN / N / NI
	2.2. **If Y/PY to 2.1**: Were the post-intervention variables that influenced selection likely to be associated with intervention?<br>2.3 **If Y/PY to 2.2**: Were the post-intervention variables that influenced selection likely to be influenced by the outcome or a cause of the outcome?		NA / Y / PY / PN / N / NI<br><br>NA / Y / PY / PN / N / NI
	2.4. Do start of follow-up and start of intervention coincide for most participants?		Y / PY / PN / N / **NI**
	2.5. **If Y/PY to 2.2 and 2.3, or N/PN to 2.4**: Were adjustment techniques used that are likely to correct for the presence of selection biases?		NA / Y / PY / PN / N / NI
	Risk of bias judgement		**Low** / Moderate / Serious / Critical / NI
	Optional: What is the predicted direction of bias due to selection of participants into the study?		**Favours experimental** / Favours comparator / Towards null /Away from null / Unpredictable
	**3. Bias in classification of interventions**		
	3.1 Were intervention groups clearly defined?	High torque group (>70Ncm), regular torque group (30-50Ncm).	Y / PY / PN / N / NI

	3.2 Was the information used to define intervention groups recorded at the start of the intervention?		Y / PY / PN / N / NI
	3.3 Could classification of intervention status have been affected by knowledge of the outcome or risk of the outcome?		Y / PY / PN / N / NI
	Risk of bias judgement		**Low** / Moderate / Serious / Critical / NI
	Optional: What is the predicted direction of bias due to classification of interventions?		**Favours experimental** / Favours comparator / Towards null /Away from null / Unpredictable
	**4. Bias due to deviations from intended interventions**		
	If your aim for this study is to assess the effect of assignment to intervention, answer questions 4.1 and 4.2		
	4.1. Were there deviations from the intended intervention beyond what would be expected in usual practice?	All interventions were performed according to clinical practice.	Y / PY / PN / N / NI
	4.2. **If Y/PY to 4.1**: Were these deviations from intended intervention unbalanced between groups *and* likely to have affected the outcome?		NA / Y / PY / PN / N / NI
	If your aim for this study is to assess the effect of starting and adhering to intervention, answer questions 4.3 to 4.6		
	4.3. Were important co-interventions balanced across intervention groups?		Y / PY / PN / N / NI
	4.4. Was the intervention implemented successfully for most participants?		Y / PY / PN / N / NI
	4.5. Did study participants adhere to the assigned intervention regimen?		Y / PY / PN / N / NI
	4.6. **If N/PN to 4.3, 4.4 or 4.5**: Was an appropriate analysis used to estimate the effect of starting and adhering to the intervention?		NA / Y / PY / PN / N / NI

	Risk of bias judgement		**Low** / Moderate / Serious / Critical / NI
	Optional: What is the predicted direction of bias due to deviations from the intended interventions?		**Favours experimental** / Favours comparator / Towards null /Away from null / Unpredictable
	**5. Bias due to missing data**		
	5.1 Were outcome data available for all, or nearly all, participants?		Y / PY / PN / N / NI
	5.2 Were participants excluded due to missing data on intervention status?		Y / PY / PN / N / NI
	5.3 Were participants excluded due to missing data on other variables needed for the analysis?		Y / PY / PN / N / NI
	5.4 **If PN/N to 5.1, or Y/PY to 5.2 or 5.3**: Are the proportion of participants and reasons for missing data similar across interventions?		NA / Y / PY / PN / N / NI
	5.5 **If PN/N to 5.1, or Y/PY to 5.2 or 5.3**: Is there evidence that results were robust to the presence of missing data?		NA / Y / PY / PN / N / NI
	Risk of bias judgement		**Low** / Moderate / Serious / Critical / NI
	Optional: What is the predicted direction of bias due to missing data?		**Favours experimental** / Favours comparator / Towards null /Away from null / Unpredictable

	**6. Bias in measurement of outcomes**		
	6.1 Could the outcome measure have been influenced by knowledge of the intervention received?		Y / PY / PN / N / NI
	6.2 Were outcome assessors aware of the intervention received by study participants?		Y / PY / PN / N / NI
	6.3 Were the methods of outcome assessment comparable across intervention groups?		Y / PY / PN / N / NI
	6.4 Were any systematic errors in measurement of the outcome related to intervention received?		Y / PY / PN / N / NI
	Risk of bias judgement		**Low** / Moderate / Serious / Critical / NI
	Optional: What is the predicted direction of bias due to measurement of outcomes?		**Favours experimental** / Favours comparator / Towards null /Away from null / Unpredictable
	**7. Bias in selection of the reported result**		
	Is the reported effect estimate likely to be selected, on the basis of the results, from...		
	7.1. ... multiple outcome *measurements* within the outcome domain?		Y / PY / PN / N / NI
	7.2 ... multiple *analyses* of the intervention-outcome relationship?		Y / PY / PN / N / NI
	7.3 ... different *subgroups*?		Y / PY / PN / N / NI
	Risk of bias judgement		**Low** / Moderate / Serious / Critical / NI

	Optional: What is the predicted direction of bias due to selection of the reported result?		**<u>Favours experimental</u>** / Favours comparator / Towards null /Away from null / Unpredictable
	**Overall bias**		
	Risk of bias judgement		**<u>Low</u>** / Moderate / Serious / Critical / NI
	Optional: What is the overall predicted direction of bias for this outcome?		**<u>Favours experimental</u>** / Favours comparator / Towards null /Away from null / Unpredictable

Table S6: Evidence of studies included in this review.

Authors and Year of Publication	Study Design and Aim	Methods	Results	Conclusions
Marconcini et al., 2018 [16]	A 3-year randomized clinical study to evaluate the role of different insertion torque values in terms of implant success, marginal bone loss, and facial soft tissues recession.	Patients requiring a single dental implant were recruited and randomized to receive one of two implants with the same macro-geometry but different cutting designs. First group consists of a 90 degrees cutting groove know as self-tapping implant, and the second group known as BlossomTM cutting groove. (Intra-Lock, Boca Raton, Florida). The insertion torque (IT) was assessed and two groups followed: high-IT (50 Ncm) group and regular-IT (<50 Ncm) group. After 3 months, all the implants were restored. At baseline, buccal bone thickness (BBT) was recorded. During the 3-year survey, the following outcomes had been registered: implant failures and success, radiographic marginal bone level around dental implant (MBL) and facial soft tissue level (FSTL).	A hundred and sixteen implants were placed in healed sites. The overall survival rate after 3 years was 96.5%. The Cumulative Success Rate was 91.3% for the High IT group and 98.2% for the Regular IT group. The mean marginal bone loss and facial soft tissue recession, at a 3-year evaluation, were significantly greater for the High-IT group and in the mandible than that reached in the Regular-IT group and in the maxilla.	Present findings showed that implants placed with higher insertion torque in mandible led to greater bone resorption and mucosal recession than that registered for implants placed with a regular IT. Moreover, sites with a thick buccal bone wall (1 mm) showed smaller recession at the facial soft tissue level after 3 years.

Barone et al., 2016 [17]	A 1-year randomized clinical trial to evaluate and compare the clinical outcome for implants placed with high insertion torque (between 50 Ncm and 100 Ncm) and regular insertion torque (within 50 Ncm) in healed ridges.	Partially edentulous patients, missing one or more mandibular or maxillary teeth, having an adequate amount of bone, requiring implant placement, were randomized to receive Blossom CT implants with regular insertion torque (<50 Ncm) or CT implants with high insertion torque (350 Ncm). Implants were left to heal submerged for 3 months. Implants were restored with individualized abutments and cemented metal-ceramic crowns. Acquired measurements were: insertion torque values (IT), thickness of buccal bone plate after implant osteotomy preparation (BBT), marginal bone level (MBL), and facial soft tissue level (FSTL). All patients were followed 12 months after implant placement.	One hundred sixteen implants were placed in one hundred sixteen patients and enrolled for the study. Fifty-eight implants were randomly allocated in *regular*-IT and *high*-IT groups with a mean insertion torque ranging from 20 Ncm to 50 Ncm and from 50 Ncm to 100 Ncm, respectively. Three implants failed, and another five implants showed at the 12-month evaluation a marginal bone loss (ΔMBL) greater than 1.5 mm, being considered unsuccessful.	The findings suggested that implants inserted with *high*-IT (350 Ncm) in healed bone ridges showed more peri-implant bone remodeling and buccal soft tissue recession than implants inserted with a *regular*-IT (<50 Ncm). Moreover, sites with a thick buccal bone wall (31 mm) – after implant osteotomy site preparation – seemed to be less prone to buccal soft tissue recession after 12 months than sites with a thin buccal bone wall (<1 mm).

Alfonsi et al., 2016 [18]	A 2-year randomized controlled clinical trial to evaluate and compare the clinical outcome for implants placed with a high insertion torque (50–100 N cm) and a regular insertion torque (within 50 N cm) in healed ridges after two years.	All of the patients were divided according to a ran- domization list into two groups: high insertion torque (CT implants insert- ed with insertion torque ≥ 50 N cm) and regular insertion torque (Blossom CT implants with insertion torque < 50 N cm). The implants were left to heal submerged for three months and then restored with individualized abutments and cemented metal–ceramic crowns. Variables registered were insertion torque values, thickness of the buccal bone plate after implant osteotomy preparation, marginal bone level and facial soft-tissue level. All of the patients were followed for two years after implant placement, with recall visits at three, six, 12 and 24 months.	116 implants were placed: 58 implants were allocated to each group, with mean insertion torque ranging from 20 N cm to 50 N cm for regular insertion torque and from 50 N cm to 100 N cm for high insertion torque. Three implants failed. Two implants showed at the 12-month evaluation a marginal bone loss > 1.5 mm and were thus considered unsuccessful.	Implants inserted with a high insertion torque in healed bone ridges showed more periimplant bone remodeling and facial soft-tissue recession than implants inserted with regular insertion torque after two years, both in the maxilla and in the mandible. The findings suggest that the clinician should pay attention to several factors in implant therapy, such as the thickness of the buccal bone, the corticalization of the surgical site, the implant's macrogeometry and the potential influence of insertion torque on implant therapy outcomes.

Hof et al., 2014 [19]	A 1-year randomized split-mouth trial to assess the impact of insertion torque and implant neck design on peri-implant bone levels and gain insights into dynamic crestal tissue alterations by radiological, clinical, and biochemical examinations.	In this prospective trial, a total of 84 implants (four implants in each patient) in the interforaminal region of 21 edentulous mandibles were randomly alternated according to a split-mouth design. Implant placement was performed using different insertion torques (220 Ncm vs >50 Ncm). In each group, one machined and one anodized implant neck design (1.5 mm length) was used in the same jaw side. Evaluation of peri-implant tissues involved radio-logical, clinical examination and immunoassays for interleukin-1b.	No significant influence of insertion torque or implant neck design on peri-implant bone level was found. Protein levels of interleukin-1b in the peri-implant crevicular fluid revealed no difference between both insertion torque groups and different neck designs.	Interactive effects of insertion torque and neck surface modification may exist; however, no clinically significant differences in marginal bone resorption after 1 year could be observed in the edentulous anterior mandible.

Makary et al., 2011 [10]	A longitudinal study to establish a correlation between dental implant insertion torque (IT) and bone density.	In 18 patients, implant site preparation was initiated using a trephine drill to retrieve a bone biopsy and completed with standard drills. Bone type was assessed during drilling according to surgeon's tactile sense. Forty implants were placed and peak IT values were recorded. Osseointegration was evaluated clinically at abutment connection. Data were analyzed using Pearson product-moment correlation coefficient.	All implants but one achieved osseointegration. D1 and D4 bone types were significantly assessed using tactile sense. IT values ranged from 15 to 150 Ncm with a mean value of 78.30 Ncm. Mean IT was significantly higher in D1 bone (126.67 Ncm) and lower in D4 bone (40.22 Ncm) (P value 0.0001), whereas in- termediate values were noted in D2 and D3 bone with no significant difference between these bone types (P value 0.462). Statistically significant correlation was found between bone volume and IT values (r 0.771, P 0.0001). No statistically significant correlation was found be- tween implant length and/or diameter and IT in all bone densities.	Clinical assessment of bone density during drilling may be achieved in hard and soft bone but not in intermediate densities. Increasing peak IT values correlated with increasing bone volume. High IT does not seem to alter osseointegration process. (Implant Dent 2011;20:182–191)

Oskouei et al., 2023 [11]	A 1-year prospective cohort study to evaluate the effect of different insertion torque values on marginal bone loss around dental implants placed in the posterior region of the mandible.	37 patients were involved in this study. Patient data (age, gender), implant characteristics (length, diameter), insertion torque values, gingival biotype, and bone quality were recorded, and parallel periapical radiographs measured marginal bone loss. The relationship between variables was obtained using independent t-tests, Pearson correlations, and regressions.	The present study found a positive and significant correlation between implant insertion torque and marginal bone loss (MBL)around the dental implants during the first year after placement. Furthermore, patients with D2 bone density at the implant placement site and thin gingival biotype also had significantly higher MBLs from baseline until crown delivery and first-year follow-up than those with D3 bone density and thick gingival biotypes, respectively.	A lower torque is necessary for high-risk patients to increase implantation success due to identifying patients with an increased risk for MBL.

Bidgoli et al., 2015 [12]	A 4-months retrospective cohort study to evaluate the effects of insertion torque on crestal bone level.	Sixty-four implants were inserted with high insertion torques (45-70 N/cm) and 72 implants were placed with a torque in the range of 20 to 30 N/cm. The distance between implant shoulder and proximal bone crest was measured on radiographs taken immediately after the insertion and compared to those calculated after un- covering surgeries (inserting the healing abutment)	The mean bone resorption around implants placed with high and lower insertion torques was 0.33 and 0.4, respectively; thus, the difference between the two groups was insignificant ($p = 0.88$).	High insertion torques (up to 70 N/cm) did not significantly increase bone resorption around implants.

Grandi et al., 2012 [13]	A 1-year multicenter controlled cohort study to evaluate the clinical outcome and the crestal bone resorption of implants placed with high insertion torque (up to 80 N cm).	102 patients were treated with 156 tapered implants. 42 implants (control group) presented insertion torque between 30 and 45 N cm (mean = 37.4 SD 8.2). 114 implants (experimental group) were placed with insertion torque between 50 and 80 N cm (mean = 74.8 SD 7.9). All implants were early loaded after 2 months. Peri-implant marginal bone levels were assessed immediately after surgery, and at 6- and 12-month follow up examinations.	At the 12-month follow up all implants were clinically stable. After 12 months, patients in the experimental group lost an average of 0.41 mm (CI 95% 0.522; 0.263) of crestal bone compared with 0.45 mm (CI 95% 0.561; 0.286) for those in the control group.	The results show that the use of high insertion torque (up to 80 N cm) did not prevent osseointegration and did not increase bone resorption around tapered implants early loaded up to 1 year after implant placement.

Aldahlawi et al., 2018 [20]	A 1-year retrospective study to evaluate and compare crestal bone levels (CBLs) after 1 year of loading of self-tapping bone condensing implants placed with high insertion torque (IT) compared to those placed with lower IT.	A retrospective chart review of 66 consecutive patients who received at least one self-tapping bone condensing implant and were in function for at least 1 year was conducted. On the basis of intrasurgical notes documenting the implant IT, the patient population was divided into group A (implant IT, >55 Ncm) and group B (IT, <55 Ncm). Radiographs taken immediately after insertion and during annual follow-up appointments were evaluated for detecting crestal bone loss. The relationship between IT and crestal bone loss, bone density, and jaw location were analyzed, and a P-value of 0.05 was considered to be statistically significant.	A total of 113 self-tapping bone condensing NobelActiveTM implants were placed. The average follow-up period from the placement of the implant restoration was 12.87 (±4.83) months. Six implants were classified as failures resulting in overall survival rate of 94.6%. Implants in group A had a mean IT of 67.35 ± 4.0 Ncm, whereas implants in the group B had a mean IT of 37.9 ± 12.62 Ncm. Implants in group A had statistically significant crestal bone loss compared to implants in group B (0.95 ± 1.60 and 0.18 ± 0.68 mm, respectively). Group A implants placed in the mandible showed significantly more pronounced crestal bone loss (2.12 ± 1.99 mm) compared to those placed in the maxilla (0.25 ± 0.65 mm; $P<0.05$); however, this was not the case in group B implants.	Implants inserted with high IT (>55 Ncm) showed more peri-implant bone remodeling than implants inserted with a less assertive IT (<55 Ncm). Bone density and jaw location affect IT and CBLs.

Rizkallah et al., 2013 [14]	A 15-months retrospective study to evaluate the correlation between insertion torque and the survival rate of immediately loaded implants placed in the maxilla.	390 NobelReplace (Nobel Biocare USA, Yorba Linda, CA) Select Tapered implants were placed in the maxillae of 145 patients over a 15-month period. The insertion torque was measured using a digital torque wrench.	Of the 390 implants, only 9 (2.3%) failed. The 381 successful implants were placed with an average insertion torque of 72.1 N.cm. Insertion torque values of failed implants varied between 45.8 and 134 N.cm, with an average of 69.2 N.cm. Of the 9 failed implants, 8 were abutments in multiple-unit implant-supported prostheses, and 1 was a single-unit implant.	There seems to be no correlation between insertion torque and implant failure above 35 N.cm threshold of insertion torque. Immediate loading of implants in the maxilla is a successful and predict- able treatment option with failure rates approaching those of traditional 2-stage surgery.

Khayat et al., 2011 [15]	A 1-year prospective study to evaluate the clinical outcome of 42 implants placed with an insertion torque equal or greater than 70Ncm and evaluate bone levels around these implants.	The study included 48 patients treated with 66 4.5 mm diameter Tapered Screw-Vent implants (Zimmer Dental®, Carlsbad, CA, USA). Maximum insertion torque (MIT) was recorded with an electronic torque measuring device (Tohnichi® STC200CN, Hitachi, Tokyo, Japan). Nine implants (control group) presented MIT between 30 and 50 Ncm (mean = 37.1 Ncm) and 42 implants (experimental group) MIT greater than 70 Ncm (mean = 110.6 Ncm, range: 70.8–176 Ncm). Marginal bone levels were recorded at the time of loading and 1 year later for the two groups.	After 2–3 months of non-sumerged healing, all implants were clinically stable. Mean marginal bone resorption was 1.03 mm (SD = 0.44) for the control group (low torque) and 0.72 mm (SD = 0.56) for the experimental group (high torque) at time of loading, and 1.09 (SD = 0.62) and 1.24 mm (SD = 0.75), respectively, after 1 year. There were no significant differences between the two groups for bone stability and implant success rate.	The use of high insertion torques (up to 176 Ncm) did not prevent osseointegration. Marginal bone levels in the control and experimental groups were similar both at the time of loading and 1 year later.

Abbreviations: BBT: buccal bone thickness; CBLs: crestal bone level FSTL: facial soft tissue level; IT: insertion torque; MBL: marginal bone level; ΔMBL: marginal bone loss; MIT: maximum insertion torque

Table S7. NHLBI Quality Assessment of Controlled Intervention Studies.

NHLBI Quality Assessment of Controlled Intervention Studies																
**First Author et al., Year**	**Q1**	**Q2**	**Q3**	**Q4**	**Q5**	**Q6**	**Q7**	**Q8**	**Q9**	**Q10**	**Q11**	**Q12**	**Q13**	**Q14**	**Total Score**	**Quality Rating**
Marconcini et al., 2018 [16]	Y	Y	Y	N	N	Y	Y	Y	Y	Y	Y	N	Y	Y	11/14 (78.57%)	Good
Barone et al., 2016 [17]	Y	Y	Y	N	Y	Y	Y	Y	Y	Y	Y	Y	Y	Y	13/14 (92.86%)	Good
Alfonsi et al., 2016 [18]	Y	Y	Y	N	N	Y	Y	Y	Y	Y	Y	N	Y	Y	11/14 (78.57%)	Good
Hof et al., 2014 [19]	Y	Y	N	N	N	Y	Y	Y	Y	Y	Y	Y	Y	Y	11/14 (78.57%)	Good

Q1: Was the study described as randomized, a randomized trial, a randomized clinical trial, or an RCT?, Q2: Was the method of randomization adequate (i.e., use of randomly generated assignment)?, Q3: Was the treatment allocation concealed (so that assignments could not be predicted)?, Q4: Were study participants and providers blinded to treatment group assignment?, Q5: Were the people assessing the outcomes blinded to the participants' group assignments?, Q6: Were the groups similar at baseline on important characteristics that could affect outcomes (e.g., demographics, risk factors, co-morbid conditions)?, Q7: Was the overall drop-out rate from the study at endpoint 20% or lower of the number allocated to treatment?, Q8: Was the differential drop-out rate (between treatment groups) at endpoint 15 percentage points or lower?, Q9: Was there high adherence to the intervention protocols for each treatment group?, Q10: Were other interventions avoided or similar in the groups (e.g., similar background treatments)?, Q11: Were outcomes assessed using valid and reliable measures, implemented consistently across all study participants?, Q12: Did the authors report that the sample size was sufficiently large to be able to detect a difference in the main outcome between groups with at least 80% power?, Q13: Were outcomes reported or subgroups analyzed prespecified (i.e., identified before analyses were conducted)?, Q14: Were all randomized participants analyzed in the group to which they were originally assigned, i.e., did they use an intention-to-treat analysis?; Total Score: Number of yes; CD: cannot be determined; NA: not applicable; NR: not reported; N: no; Y: yes. Quality Rating: Poor <50%, Fair 50–75%, Good ≥75%.

Table S8. NHLBI Quality Assessment Tool for Observational Cohort and Cross-Sectional Studies.

	NHLBI Quality Assessment Tool for Observational Cohort and Cross-Sectional Studies														
**First Author et al., Year**	**Q1**	**Q2**	**Q3**	**Q4**	**Q5**	**Q6**	**Q7**	**Q8**	**Q9**	**Q10**	**Q11**	**Q12**	**Q13**	**Q14**	**Total Score**	**Quality Rating**
Makary et al., 2011 [10]	Y	Y	NR	Y	N	Y	Y	N	Y	N	Y	N	NA	Y	8/14 (57.14%)	Fair
Oskouei et al., 2023 [11]	Y	Y	NR	Y	Y	Y	Y	N	Y	Y	Y	N	N	Y	10/14 (71.42%)	Good
Bidgoli et al., 2015 [12]	Y	Y	NR	Y	N	Y	Y	N	Y	Y	Y	Y	Y	Y	11/14 (78.57%)	Good
Grandi et al., 2012 [13]	Y	Y	NR	Y	N	Y	Y	N	Y	Y	Y	N	Y	Y	10/14 (71.42%)	Good
Rizkallah et al., 2013 [14]	Y	Y	NR	N	N	Y	Y	N	Y	Y	Y	N	Y	Y	10/14 (71.42%)	Good
Khayat et al, 2011 [15]	Y	Y	NR	Y	N	Y	Y	N	Y	Y	Y	N	Y	Y	10/14 (71.42%)	Good
Aldahlawi et al., 2017 [20]	Y	Y	NR	Y	N	Y	Y	N	Y	Y	Y	N	Y	Y	10/14 (71.42%)	Good

Q1: Was the research question or objective in this paper clearly stated?, Q2: Was the study population clearly specified and defined?, Q3: Was the participation rate of eligible persons at least 50%?, Q4: Were all the subjects selected or recruited from the same or similar populations (including the same time period)? Were inclusion and exclusion criteria for being in the study prespecified and applied uniformly to all participants?, Q5: Was a sample size justification, power description, or variance and effect estimates provided?, Q6: For the analyses in this paper, were the exposure(s) of interest measured prior to the outcome(s) being measured?, Q7: Was the timeframe sufficient so that one could reasonably expect to see an association between exposure and outcome if it existed?, Q8: For exposures that can vary in amount or level, did the study examine different levels of the exposure as related to the outcome (e.g., categories of exposure, or exposure measured as continuous variable)?, Q9: Were the exposure measures (independent variables) clearly defined, valid, reliable, and implemented consistently across all study participants?, Q10: Was the exposure(s) assessed more than once over time?, Q11: Were the outcome measures (dependent variables) clearly defined, valid, reliable, and implemented consistently across all study participants?, Q12: Were the outcome assessors blinded to the exposure status of participants?, Q13: Was loss to follow-up after baseline 20% or less?, Q14: Were key potential confounding variables measured and adjusted statistically for their impact on the relationship between exposure(s) and outcome(s)?; Total Score: Number of yes; CD: cannot be determined; NA: not applicable; NR: not reported; N: no; Y: yes. Quality Rating: Poor <50%, Fair 50–75%, Good

References

1. Li H., Liang Y., Zheng Q. Meta-Analysis of Correlations Between Marginal Bone Resorption and High Insertion Torque of Dental Implants. Int J Oral Maxillofac Implants. 2015;30(4):767–772.
2. Roca-Millan E., González-Navarro B., Domínguez-Mínger J., Marí-Roig A., Jané-Salas E., López-López J. Implant insertion torque and marginal bone loss: A systematic review and meta-analysis. Int J Oral Implantol (Berl). 2020;13(4):345–353.
3. Atieh M.A., Baqain Z.H., Tawse-Smith A., Ma S., Almoselli M., Lin L., Alsabeeha N.H.M. The influence of insertion torque values on the failure and complication rates of dental implants: A systematic review and meta-analysis. Clin Implant Dent Relat Res. 2021;23(3):341–360.
4. Darriba I., Seidel A., Moreno F., Botelho J., Machado V., Mendes J.J., Leira Y., Blanco J. Influence of low insertion torque values on survival rate of immediately loaded dental implants: A systematic review and meta-analysis. J Clin Periodontol. 2023;50(2):158–169.
5. Berardini M., Trisi P., Sinjari B., Rutjes A.W., Caputi S. The Effects of High Insertion Torque Versus Low Insertion Torque on Marginal Bone Resorption and Implant Failure Rates: A Systematic Review With Meta-Analyses. Implant Dent. 2016;25(4):532–540.
6. Di Stefano D.A., Piattelli A., Iezzi G., Orlando F., Arosio P. Cortical Thickness, Bone Density, and the Insertion Torque/Depth Integral: A Study Using Polyurethane Foam Blocks. Int J Oral Maxillofac Implants. 2021;36(3):423–431.
7. Ikar M., Grobecker-Karl T., Karl M., Steiner C. Mechanical stress during implant surgery and its effects on marginal bone: a literature review. Quintessence Int. 2020;51(2):142–150.
8. Lemos C.A.A., Verri F.R., de Oliveira Neto O.B., Cruz R.S., Luna Gomes J.M., da Silva Casado B.G., Pellizzer E.P. Clinical effect of the high insertion torque on dental implants: A systematic review and meta-analysis. J Prosthet Dent. 2021;126(4):490–496.
9. Radi I.A., Samy M.A.E.Y. Limited evidence suggests no correlation between implant insertion torque and implant survival and marginal bone loss. J Evid Based Dent Pract. 2023;23:101839.
10. Makary C., Rebaudi A., Mokbel N., Naaman N. Peak insertion torque correlated to histologically and clinically evaluated bone density. Implant Dent. 2011;20(3):182–191.
11. Oskouei A.B., Golkar M., Badkoobeh A., Jahri M., Sadeghi H.M.M., Mohammadikhah M., Abbasi K., Tabrizi R., Alam M. Investigating the effect of insertion torque on marginal bone loss around dental implants. J Stomatol Oral Maxillofac Surg. 2023;124(6S):101523.
12. Bidgoli M., Soheilifar S., Faradmal J., Soheilifar S. High Insertion Torque and Peri-Implant Bone Loss: Is There a Relationship? J Long Term Eff Med Implants. 2015;25(3):209–213.
13. Grandi T., Guazzi P., Samarani R., Grandi G. Clinical outcome and bone healing of implants placed with high insertion torque: 12-month results from a multicenter controlled cohort study. Int J Oral Maxillofac Surg. 2013;42(4):516–520.
14. Rizkallah N., Fischer S., Kraut R.A. Correlation between insertion torque and survival rates in immediately loaded implants in the maxilla: a retrospective study. Implant Dent. 2013;22(3):250–254.
15. Khayat P.G., Arnal H.M., Tourbah B.I., Sennerby L. Clinical outcome of dental implants placed with high insertion torques (up to 176 Ncm). Clin Implant Dent Relat Res. 2013;15(2):227–233.
16. Marconcini S., Giammarinaro E., Toti P., Alfonsi F., Covani U., Barone A. Longitudinal analysis on the effect of insertion torque on delayed single implants: A 3-year randomized clinical study. Clin Implant Dent Relat Res. 2018;20(3):322–332.
17. Barone A., Alfonsi F., Derchi G., Tonelli P., Toti P., Marchionni S., Covani U. The Effect of Insertion Torque on the Clinical Outcome of Single Implants: A Randomized Clinical Trial. Clin Implant Dent Relat Res. 2016;18(3):588–600.

18. Alfonsi F., Borgia V., Barbato L., Tonelli P., Giammarinaro E., Marconcini S., Romeggio S., Barone A. The clinical effects of insertion torque for implant placed in healed ridges: a two-year randomized controlled clinical trial. J Oral Sci Rehabil. 2016;2(4):62–73.
19. Hof M., Pommer B., Strbac G.D., Vasak C., Agis H., Zechner W. Impact of insertion torque and implant neck design on peri-implant bone level: a randomized split-mouth trial. Clin Implant Dent Relat Res. 2014;16(5):668–674.
20. Aldahlawi S., Demeter A., Irinakis T. The effect of implant placement torque on crestal bone remodeling after 1 year of loading. Clin Cosmet Investig Dent. 2018;10:203–209.
